# SARS-CoV-2 nucleocapsid protein directly prevents cGAS–DNA recognition through competitive binding

**DOI:** 10.1073/pnas.2426204122

**Published:** 2025-06-23

**Authors:** Theresia Gutmann, David Kuster, Anthony A. Hyman

**Affiliations:** ^a^Max Planck Institute of Cell Biology and Genetics, Dresden 01307, Germany

**Keywords:** cGAS, nucleocapsid protein, biomolecular condensates, innate immune system, coronavirus

## Abstract

A hallmark of severe acute respiratory syndrome coronavirus 2 (SARS-CoV-2) infection is the delayed interferon response. Interferons are typically produced upon host recognition of pathogen- or damage-associated molecular patterns, such as nucleic acids. While the mechanisms by which SARS-CoV-2 evades host recognition of its RNA are well studied, how it evades immune responses to cytosolic DNA—leaked from mitochondria or nuclei during infection—remains poorly understood. Here, we demonstrate that the SARS-CoV-2 nucleocapsid protein directly suppresses DNA sensing by cyclic guanosine monophosphate–adenosine monophosphate synthase (cGAS). Although primarily known for packaging the viral RNA genome, we uncover that the SARS-CoV-2 nucleocapsid protein also binds DNA with high affinity and competitively blocks cGAS activation. Using cell-free biochemical and biophysical approaches, including single-molecule optical tweezers, we show that the nucleocapsid protein binds to DNA at nanomolar concentrations and cocondenses with DNA at micromolar concentrations, thereby impeding stable cGAS-DNA interactions required for signal propagation. Hyperphosphorylation of the nucleocapsid protein diminishes its competitive binding capacity. Our findings reveal an unexpected role of the SARS-CoV-2 nucleocapsid protein in directly suppressing the cGAS-STING pathway, strongly suggesting that this contributes to the delayed interferon response during infection. This study raises the possibility that nucleocapsid proteins of other RNA viruses may also exhibit moonlighting functions by antagonizing host nucleic acid–sensing pathways.

## Significance

Severe acute respiratory syndrome coronavirus 2 (SARS-CoV-2) is a master of immune evasion, employing multiple mechanisms to subvert host immune responses. While its ability to block RNA-sensing pathways is well established, how SARS-CoV-2 prevents the recognition of cytosolic DNA—leaked from mitochondria or nuclei during infection—remains poorly understood. This study reveals that the SARS-CoV-2 nucleocapsid protein, primarily known for packaging the RNA genome, also antagonizes DNA sensing by cyclic guanosine monophosphate–adenosine monophosphate synthase (cGAS). By competitively binding and condensing DNA, the nucleocapsid protein disrupts cGAS–DNA interactions, impairing immune signaling. These findings may point to a broader paradigm in which RNA virus nucleocapsid proteins regulate both RNA- and DNA-sensing pathways, with potential implications for the development of antiviral therapeutics.

Severe acute respiratory syndrome coronavirus 2 (SARS-CoV-2) has rapidly disseminated worldwide, causing the COVID-19 pandemic and reshaping global health and society (1, 2). Its remarkable transmission and replication efficiency has been largely attributed to its ability to interfere with the host immune system (3). In the early stages of infection, SARS-CoV-2 suppresses innate immune responses in infected cells, leading to a delayed induction of type I and III interferons (3–5). These interferons, critical for restricting viral replication, are typically produced in response to the recognition of pathogen- or damage-associated molecular patterns, including viral nucleic acids or mislocalized host DNA. As SARS-CoV-2 contains an RNA genome, most research has focused on its ability to evade RNA-sensing pathways of the innate immune system (3). In contrast, how the virus suppresses interferon production in response to cytosolic host DNA is poorly understood.

Cytosolic host DNA is thought to originate from the leakage of host mitochondrial or nuclear DNA into the cytosol of SARS-CoV-2-infected cells, likely resulting from mitochondrial stress and extensive virus-induced host endomembrane remodeling (6, 7). This would typically activate sensors of the innate immune system (8); however, during early stages of SARS-CoV-2 infection, this immune response is suppressed. The major immune sensor for such mislocalized DNA is cyclic guanosine monophosphate–adenosine monophosphate synthase (cGAS). cGAS binds DNA in a sequence-independent manner, with a preference for long DNA, and forms clusters that assemble into phase-separated compartments (9–11). Upon activation by DNA, cGAS converts the nucleotides adenosine triphosphate (ATP) and guanosine triphosphate (GTP) to 2′-3′-cyclic GMP-AMP (cGAMP), which induces the stimulator of interferon genes (STING) to trigger the production of type I or III interferons and proinflammatory cytokines (12, 13).

During the late stages of COVID-19, mislocalized host DNA drives excessive interferon responses via the cGAS–STING pathway (14–18). In contrast, at the onset of the disease, the cGAS-mediated interferon response is typically blunted, which allows for high viral loads in patients (3). This suggests that viral factors antagonize cGAS signaling, yet the mechanisms by which SARS-CoV-2 initially disrupts cytosolic DNA sensing remain understudied.

One candidate for this interference is the SARS-CoV-2 nucleocapsid protein, which rapidly becomes one of the most abundant proteins in infected cells (19–21). As a conserved structural viral component, the nucleocapsid protein compacts the ~30-kb viral RNA genome to facilitate virion assembly (22). Phosphorylation regulates the nucleocapsid protein’s ability to bind and compact RNA, as well as its propensity to oligomerize and form biomolecular condensates (23–26). Beyond its role in RNA genome packaging, the nucleocapsid protein participates in viral transcription (27–29) and has been observed to suppress innate immune responses, partly by its interference with RNA-sensing pathways (16, 30–33).

In this study, we reveal a potential immune evasion mechanism whereby the SARS-CoV-2 nucleocapsid protein directly prevents cGAS activation by competitively binding or condensing DNA, a process that depends on the phosphorylation status of the nucleocapsid protein. By this mechanism, the SARS-CoV-2 nucleocapsid protein would contribute to the repertoire of immune evasion strategies, enabling the virus to antagonize cytosolic DNA detection in infected cells efficiently.

## Results

### Purification and Characterization of cGAS and SARS-CoV-2 Nucleocapsid Proteins.

**Fig. 1.**
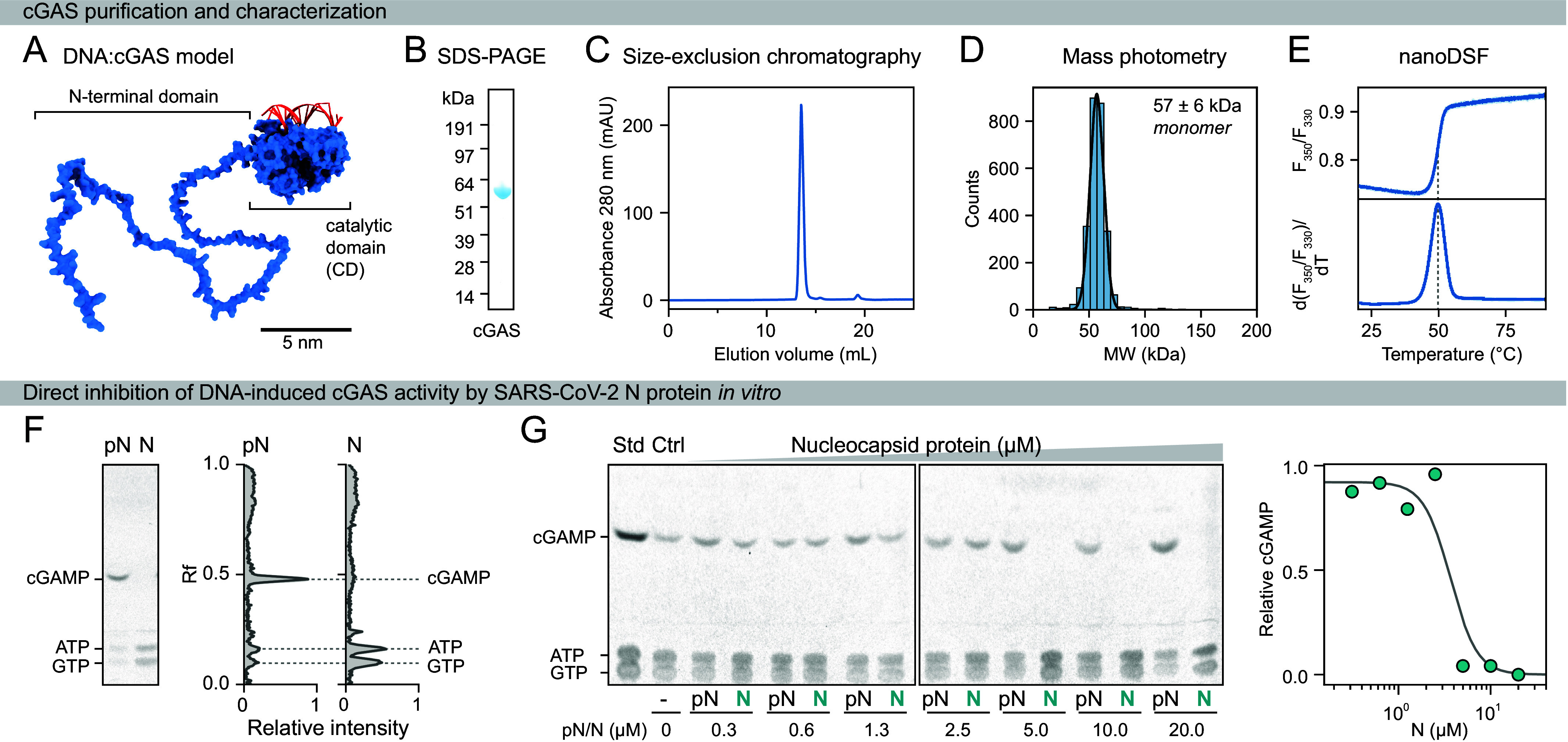
Characterization of recombinant cGAS and inhibition of its activity by the SARS-CoV-2 nucleocapsid protein. (*A*) Structural model of full-length human cGAS (blue) bound to 17-bp DNA (red) at the primary DNA-binding site, based on the crystal structure of the catalytic domain [PDB: 6CT9, (34)]. The disordered N-terminal domain was modeled using AlphaFold, with torsion angles adjusted for optimal visualization. (*B*) Coomassie-stained SDS-PAGE gel of purified human cGAS. (*C*) Size-exclusion chromatography confirms the homogeneity of recombinant cGAS, which elutes as a monomer from a Superdex 200 Increase 10/300 GL column. (*D*) Mass photometry analysis indicated a molecular weight of 59 ± 7.5 kDa, consistent with monomeric full-length cGAS. (*E*) The thermal stability of cGAS was measured by nanoDSF, which assessed the intrinsic fluorescence ratio at 350 and 330 nm (n = 2; error bands represent SD). A dashed line indicates the melting temperature of 49.80 ± 0.04 °C. (*F*) In vitro synthesis of 2′3′-cGAMP by cGAS in the presence of 0.2 µM 100-bp DNA and 10 µM unphosphorylated (N) or phosphorylated (pN) nucleocapsid protein in 120 mM NaCl, 20 mM HEPES, pH 7.5, 5 mM MgCl_2_, 0.5 mM ATP, 0.5 mM GTP, and 1 mM DTT. The thin-layer chromatography plate shows separated nucleotides (*Left*) with the corresponding densitograms (*Right*). (*G*) Dose-dependent inhibition of cGAS (1 µM) activity in the presence of 0.2 µM 100-bp DNA and increasing concentrations of N or pN protein. The *Right* panel shows cGAMP production relative to the nucleocapsid protein-free control.

To investigate the impact of SARS-CoV-2 nucleocapsid proteins on DNA-induced cGAS activity, we employed a cell-free approach and reconstituted cGAS activation in vitro in the presence of nucleocapsid protein assemblies using purified components. We first expressed the full-length human cGAS and the SARS-CoV-2 nucleocapsid protein in insect cells using baculoviral vectors. All proteins were purified using a three-step chromatographic procedure and were free of detectable nucleic acid contaminants (Fig. 1 and *SI Appendix*, Figs. S1 and S2). Fluorescent or AviTag fusion proteins were produced analogously to the respective label-free protein variants. Biochemical and biophysical analyses confirmed the homogeneity, purity, and conformational thermostability of the proteins (Fig. 1 and *SI Appendix*, Figs. S1 and S2). Recombinant cGAS is monomeric, as validated by size-exclusion chromatography coupled to static light scattering and mass photometry, and exhibits DNA-dependent nucleotidyltransferase activity (Fig. 1 and *SI Appendix*, Fig. S1).

The SARS-CoV-2 nucleocapsid protein, recombinantly produced in Sf9 insect cells, forms a hyperphosphorylated dimer, hereafter referred to as phospho-nucleocapsid (pN) protein (*SI Appendix*, Fig. S2). Intact protein mass spectrometry revealed a high degree of phosphorylation with up to 15 phosphoryl groups (*Materials and Methods* and *SI Appendix*, Fig. S2*I*). Hyperphosphorylated nucleocapsid protein states have been observed in SARS-CoV-2-infected mammalian cells (35), where phosphorylation regulates nucleocapsid protein self-association and binding to viral RNA—functions essential for transcription and viral genome packaging (23–26). To investigate whether the phosphorylation state impacts cGAS activation, we also purified the dephosphorylated nucleocapsid (N) protein dimer by introducing a λ-phosphatase treatment during purification. Complete dephosphorylation was confirmed by mass spectrometry (*SI Appendix*, Fig. S2*I*).

The SARS-CoV-2 nucleocapsid protein exhibits a strong propensity to self-associate, a feature that is modulated by its phosphorylation status. Owing to this self-association tendency and its high abundance in infected cells, the protein was suggested to form biomolecular condensates during infection (19–21, 35–37). In agreement with previous studies [reviewed in Cascarina and Ross (26)], we observed that phosphorylation alters the nucleocapsid protein’s self-association and condensation behavior (*SI Appendix*, Fig. S2*H*). The unphosphorylated N protein readily formed homotypic condensates in the presence of divalent Mg^2+^ ions at physiological ionic strength (up to 196 mM NaCl)—observable as protein droplets by fluorescence microscopy. In contrast, the pN protein formed condensates only at lower ionic strength (<70 mM NaCl), indicating reduced self-association under physiological conditions.

### SARS-CoV-2 N Protein Antagonizes cGAS Activity via Competitive Binding to Short DNA Fragments.

Upon binding to double-stranded DNA, cGAS is allosterically activated to produce the cyclic dinucleotide cGAMP from the nucleotides ATP and GTP (*SI Appendix*, Fig. S1*G*). To investigate whether and how the SARS-CoV-2 nucleocapsid proteins influence cGAS activity, we measured cGAMP production in the presence of 100-bp double-stranded DNA (100-bp DNA) at varying nucleocapsid protein concentrations using thin-layer chromatography. In the presence of DNA—providing stoichiometric cGAS binding sites [assuming a 20-bp cGAS footprint (38)]—the N protein abolished cGAS nucleotidyltransferase activity in a dose-dependent manner when present in stoichiometric excess (Fig. 1 *F* and *G*). In contrast, the pN protein did not affect cGAMP production under any of the tested conditions (Fig. 1 *F* and *G*). cGAS activity was restored by adding excess DNA to the reaction, suggesting that competitive DNA binding by the nucleocapsid protein accounts for the observed inhibition of cGAS (*SI Appendix*, Fig. S3). Notably, under these enzymatic assay buffer conditions containing 120 mM NaCl, the N protein readily forms biomolecular condensates, whereas the pN protein does not.

The ability of the N protein to condense might enhance its inhibitory effect on cGAS activityand contribute to the steep slope of the observed dose–response curves (Fig. 1*G* and *SI Appendix*, Fig. S3*A*). Given that both cGAS and the N protein possess multivalent binding interfaces and that the DNA itself can scaffold multiple binding events, cooperative mechanisms are highly plausible. Indeed, both cGAS and the N protein are known to exhibit cooperative nucleic acid binding and multimerization (38, 39). Furthermore, N protein binding potentially alters the DNA conformation, thereby impeding cGAS binding, which is sensitive to DNA structure (38, 40, 41). However, while our data indicate cooperativity, the near-saturating assay conditions render the system particularly susceptible to ligand depletion effects and preclude the extraction of interaction parameters.

**Fig. 2.**
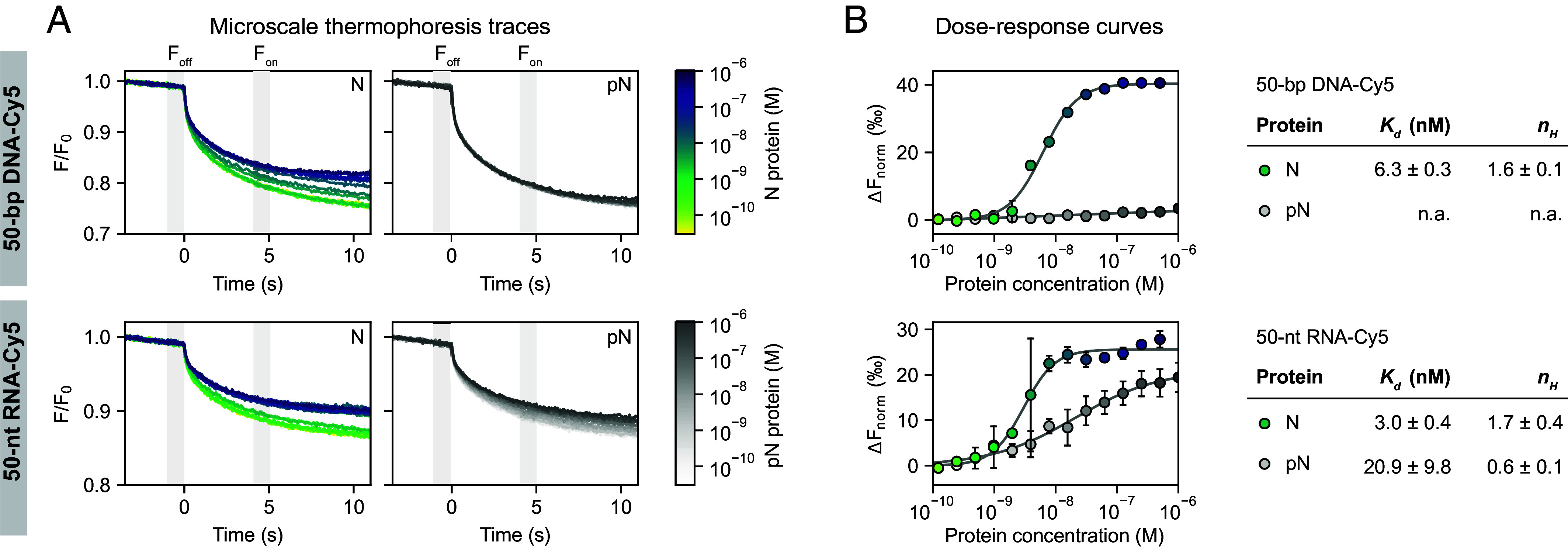
SARS-CoV-2 N protein binds to DNA and RNA with high affinity. Microscale thermophoresis was used to characterize nucleic acid binding by nucleocapsid proteins in a buffer containing 20 mM HEPES, pH 7.5, 150 mM NaCl, 0.05% Tween-20, and 1 mM DTT. (*A*) Normalized microscale thermophoresis traces show the binding of label-free N and pN proteins to 0.75 nM of Cy5-labeled 50-bp DNA (*Top*) and 50-nt RNA (*Bottom*). Gray-shaded time intervals were analyzed to generate dose–response curves shown in (*B*). Each trace is color-coded by protein concentration. (*B*) Corresponding dose–response curves for N and pN protein binding to DNA (*Top*) and RNA (*Bottom*) were generated by fitting the change in thermophoretic mobility to the Hill equation. Points represent the mean of two independent experiments, color-coded as in (*A*), with error bars representing SD. Apparent dissociation constants (K_d_) and Hill coefficients (n_H_) are reported ± SD.

To distinguish effects of nucleic acid binding from those of condensation, we performed binding affinity and competition assays under conditions that do not promote phase separation—specifically, at elevated ionic strength (150 mM NaCl) and reduced protein concentrations (≤1 µM). We determined the apparent binding affinity of the nucleocapsid proteins for nucleic acid fragments in solution by microscale thermophoresis using Cy5-labeled 50-nt RNA or 50-bp DNA oligonucleutides (Fig. 2). As expected for an RNA-binding protein, the N protein has a high apparent affinity for RNA and binds with moderate positive cooperativity (K_d_ = 3.0 ± 0.4 nM; n_H_ = 1.7 ± 0.4), both of which are decreased by phosphorylation (K_d_ = 20.9 ± 9.8 nM; n_H_ = 0.6 ± 0.1). Notably, the unphosphorylated N protein also binds to DNA fragments with a surprisingly high apparent affinity (K_d_ = 6.3 ± 0.3 nM) and moderate positive cooperativity (n_H_ = 1.6 ± 0.1). In contrast, no binding was observed for the hyperphosphorylated form.

We further demonstrated the N protein’s competitive DNA binding using biolayer interferometry, where the biotinylated cGAS catalytic domain (biotin-AviTag-cGAS^CD^) was immobilized on a streptavidin sensor (*SI Appendix*, Fig. S4). After immobilization, we incubated the sensor with DNA fragments at a saturating concentration. We then exposed the biotin-AviTag-cGAS^CD^:DNA complexes to N protein solutions of increasing concentrations while monitoring the association and dissociation from the sensor. The N protein markedly accelerated the unbinding of the 20-bp DNA from the sensor in a concentration-dependent manner (*SI Appendix*, Fig. S4*A*). After binding longer 45-bp DNA to the immobilized cGAS^CD^, we first observed an N protein concentration-dependent increase in the binding signal, followed by accelerated dissociation, suggesting that the N protein first binds to unoccupied sites on the DNA bound to immobilized cGAS^CD^ before competing cGAS^CD^ away and dissociating together with the DNA from the sensor (*SI Appendix*, Fig. S4*B*).

In summary, the unmodified SARS-CoV-2 N protein binds short DNA fragments with high affinity and outcompetes cGAS binding at nanomolar concentrations.

### DNA–cGAS Interactions in the Context of Nucleocapsid Protein Condensates and Emergent Assembly Properties.

**Fig. 3.**
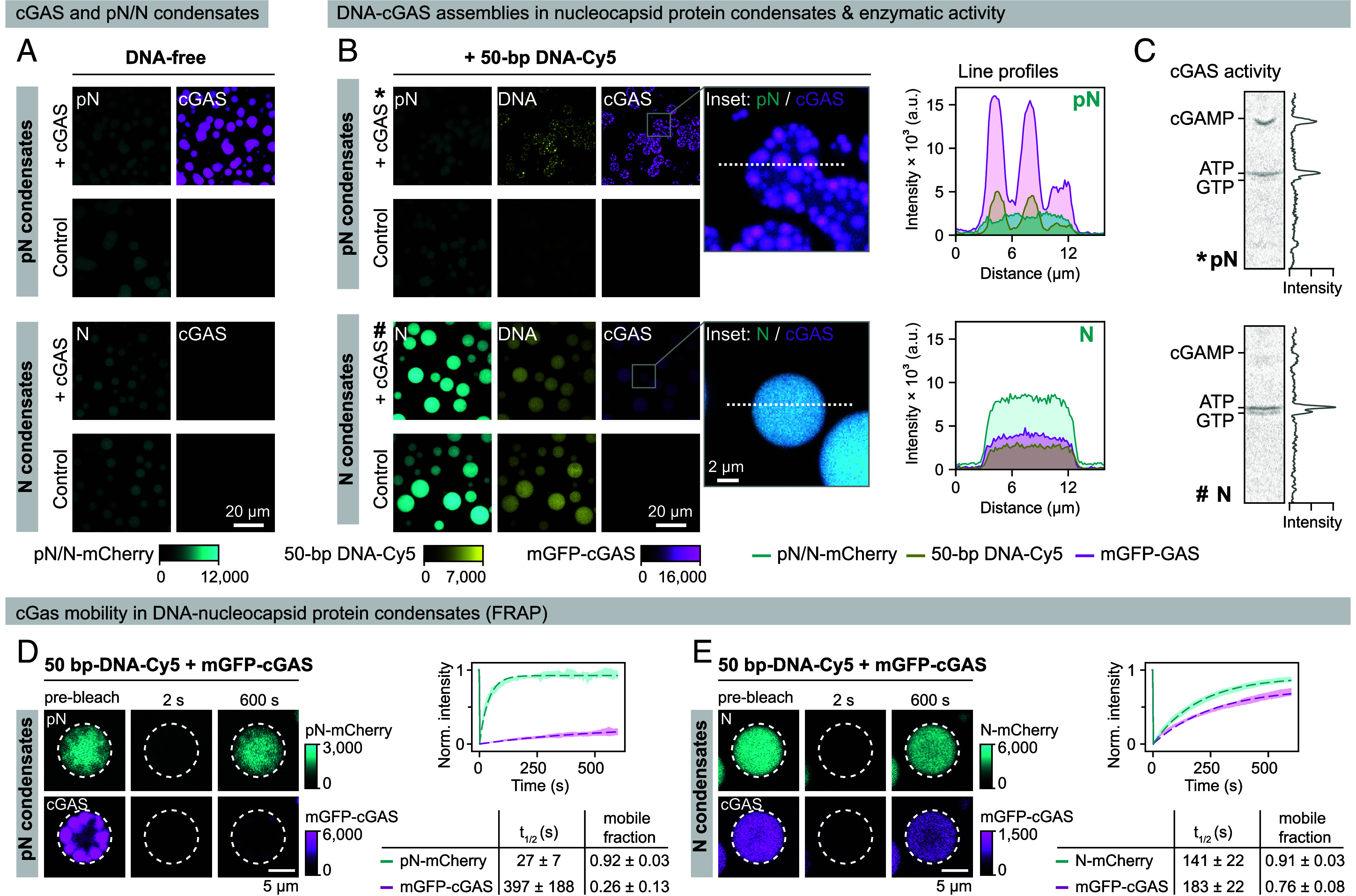
DNA–cGAS clusters do not form within nucleocapsid protein condensates. Condensates were formed by mixing pN or N protein (10 µM, including 10 mol% of the respective mCherry-labeled nucleocapsid) in a buffer containing 50 mM NaCl, 20 mM HEPES, pH 7.5, 5 mM MgCl_2_, 0.1 mM ATP, 0.1 mM GTP, 5% glycerol, 2 mM DTT; in the presence or absence of mGFP-cGAS (1 µM) and/or 50-bp DNA-Cy5 (0.5 µM). (*A*) Confocal microscopy images showing partitioning of mGFP-cGAS into DNA-free pN (*Top*) and N protein (*Bottom*) condensates. mGFP-cGAS partitions preferentially into pN protein condensates. (*B*) Confocal microscopy images showing partitioning of mGFP-cGAS into DNA-containing pN (*Top*) and N protein (*Bottom*) condensates. DNA has a high affinity for the unphosphorylated N protein and enhances N protein condensation and protein density in the condensed phase. Magnified merged images on the right demonstrate the DNA–cGAS clustering in pN versus homogeneous partitioning in N protein condensates. Fluorescence signal intensities along the dashed lines are shown in the graphs. (*C*) cGAS activity in the presence of DNA and pN (*) or N protein condensates (#) monitored by thin-layer chromatography. (*D* and *E*) FRAP analysis reveals differential mGFP-cGAS mobility in the presence of DNA and pN (*D*) or N protein condensates (*E*). Panels show representative snapshots before, at 2 s, and at 600 s after bleaching. Fluorescence intensities were fitted to a diffusion model. Graphs show the fitted curves (dashed line), and the error band represents the mean intensities ± SD (n = 7 droplets). mGFP-cGAS forms immobile clusters in the presence of pN protein condensates and retains mobility within N-DNA condensates.

We next sought to determine whether nucleocapsid protein condensates affect cGAS activation. To this end, we used conditions that promote condensate formation by both N and pN proteins and are, in principle, compatible with cGAS activity. We first induced homotypic nucleocapsid protein condensates in the presence of Mg^2+^ ions and ATP/GTP nucleotides by lowering the salt concentration to 50 mM NaCl and using nucleocapsid protein concentrations of 10 µM (Fig. 3*A* and *SI Appendix*, Fig. S5 *A* and *B*). We assessed protein partitioning and dynamics by confocal fluorescence microscopy and subsequently determined cGAMP production by thin-layer chromatography. For visualization, we used an mGFP-cGAS fusion protein (mGFP-cGAS), Cy5-labeled oligonucleotides, and nucleocapsid proteins spiked with 10 mol% of the corresponding mCherry-tagged variant (N-mCherry or pN-mCherry).

In the absence of nucleic acids, cGAS preferentially partitioned into pN protein condensates, presumably reflecting transient nonspecific electrostatic interactions between the highly positively charged cGAS (predicted isoelectric point of 9.5) and the negatively charged phosphate groups of the pN protein (Fig. 3*A* and *SI Appendix*, Fig. S5). As expected, cGAS remains inactive without DNA and is not activated by other types of nucleic acid fragments, such as single-stranded DNA or RNA (*SI Appendix*, Figs. S1*F* and S2*J*). Only in the presence of double-stranded DNA was cGAS activated to catalyze cGAMP production (Fig. 3*C*).

Consistent with our previous experiments, DNA-induced cGAMP production was unaffected by the presence of pN protein condensates. However, it was completely inhibited in the presence of N protein (Fig. 3 *B* and *C*). This difference was reflected in the material properties of the phase-separated assemblies: In the presence of pN protein condensates, cGAS cocondensed with DNA into highly concentrated clusters that appeared as phase-separated foci associated with pN protein condensates (Fig. 3*B*). Within these clusters, cGAS mobility was low as demonstrated by fluorescence recovery after photobleaching (FRAP) experiments, which showed only 26 ± 13% signal recovery within 600 s, compared to the highly mobile pN protein (mobile fraction = 92 ± 3%, t_1/2_ = 27 ± 7 s) (Fig. 3*D*). In contrast, cGAS copartitioned homogeneously with DNA into N protein condensates (Fig. 3 *B* and *E* and *SI Appendix*, Fig. S5*C*), where it maintained substantially higher mobility (76 ± 8% mobile fraction, t_1/2_ = 183 ± 22 s), while the N protein showed an increased half-time of recovery (141 ± 22 s).

To confirm the Cy5 label did not influence partitioning and binding, we formed nucleocapsid protein or cGAS condensates in the presence of either Cy5-labeled or label-free 50-bp DNA. We analyzed the DNA fraction in the dilute phase by denaturing polyacrylamide gel electrophoresis (*SI Appendix*, Fig. S6). Both labeled and unlabeled DNA oligos exhibited similar behavior, with comparable levels of free DNA and urea-resistant complex formation with the respective proteins. Notably, across all DNA concentrations tested (0.25, 0.5, and 1.0 µM), no free DNA was detectable in the N protein samples, highlighting its strong propensity to bind and condense DNA. In contrast, pN and cGAS samples retained a distinct pool of unbound DNA in the dilute phase. These results further support the conclusion that unphosphorylated N protein robustly interacts with DNA and efficiently sequesters it into condensates.

In conclusion, the impact of N protein on cGAS-DNA interactions was also apparent within biomolecular nucleocapsid protein condensates (Fig. 3 and *SI Appendix*, Fig. S5). Our data suggest that even though cGAS can partition into DNA-containing N protein condensates, it likely fails to form sufficiently stable interactions with DNA. This may prevent the enzyme from reaching the local concentrations or the minimal residence time on DNA required for its productive activation.

### Visualizing N Protein Interference with cGAS Binding to Long DNA Using Optical Tweezers.

**Fig. 4.**
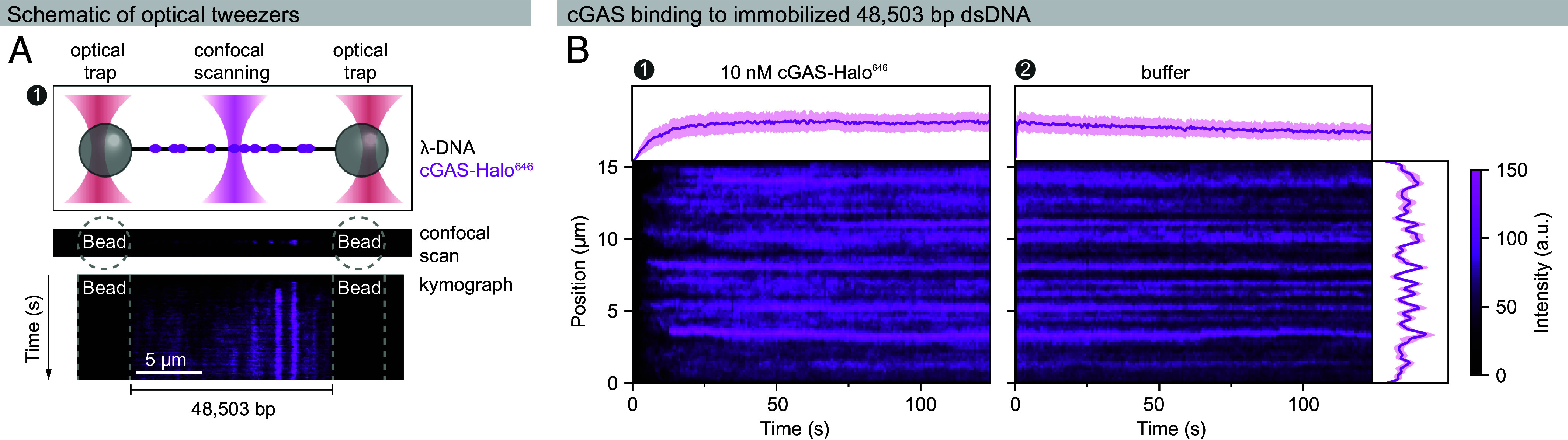
Real-time visualization of cGAS binding to long DNA using single-molecule optical tweezers. The dynamic binding and unbinding of cGAS-Halo^646^ to a single DNA molecule were monitored using optical tweezers. Experiments were conducted in a buffer containing 50 mM NaCl, 20 mM HEPES, pH 7.0, 5% glycerol, 2 mM DTT, and 0.3 mg/mL BSA. (*A*) Schematic presenting the optical tweezer setup used to study cGAS-DNA interactions. Biotinylated λ-phage DNA (black) is tethered between two optically trapped, streptavidin-functionalized polystyrene beads. The DNA is exposed to cGAS-Halo^646^, which binds and forms clusters along the DNA (magenta). Confocal microscopy is used to scan the DNA tether. Kymograph projections along the DNA visualize the spatiotemporal dynamics of protein-DNA interactions. (*B*) Representative kymographs show cGAS-Halo^646^ interactions with DNA at 10 nM (*Left*) and after transferring the DNA tether to buffer (*Right*). The corresponding fluorescence intensity profiles are plotted over time (above kymographs) or along the DNA (*Left*), representing mean pixel intensity ± SD. cGAS-Halo^646^ binds rapidly to DNA and forms stable clusters that persist after the DNA tether is transferred to plain buffer, reflecting the multivalent interactions and resulting high avidity within cGAS-DNA clusters.

The binding of cGAS to DNA—as well as its condensation and activation—is sensitive to the DNA length: binding affinity and enzymatic activity increase with longer DNA molecules (9, 38, 42). To investigate how nucleocapsid proteins affect cGAS interactions with long DNA, we employed a dual-trap optical tweezers system to visualize binding to a single λ-phage DNA molecule (48,503 bp) tethered between two streptavidin-functionalized polystyrene beads (Fig. 4*A*). This approach enabled real-time, single-molecule measurements of protein–DNA interactions while preventing intermolecular DNA aggregation. For visualization, we used a HaloTag-labeled cGAS fusion protein (cGAS-Halo^646^) and GFP-tagged nucleocapsid proteins (mGFP-N or mGFP-pN).

In the absence of nucleocapsid proteins, cGAS-Halo^646^ readily bound to DNA at a concentration of 10 nM, reaching saturation within ~30 s (Fig. 4*B*). cGAS formed highly enriched clusters on DNA that remained stable even after the DNA molecule was transferred back into plain buffer, presumably reflecting the multivalent DNA binding of cGAS and the resulting avidity effects within the clusters (Fig. 4*B*).

**Fig. 5.**
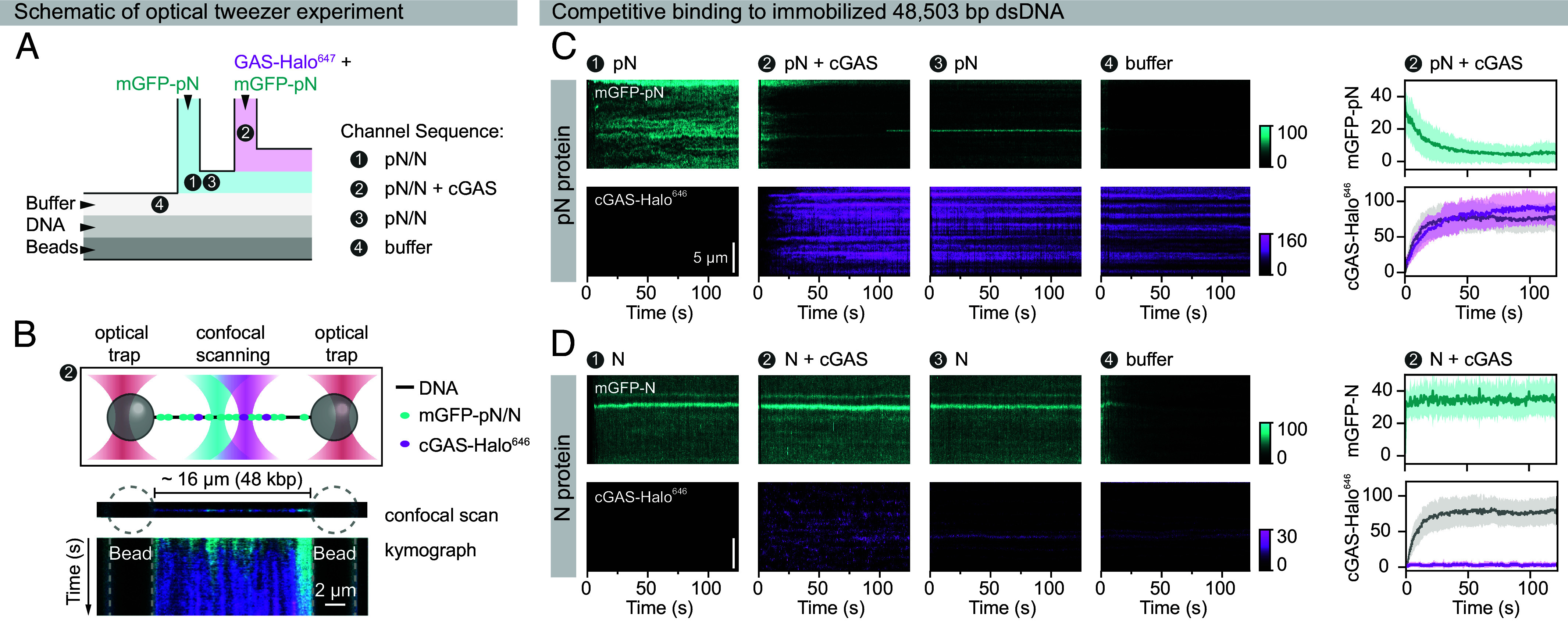
Competitive binding of SARS-CoV-2 nucleocapsid proteins to long DNA revealed by single-molecule optical tweezer assays. Simultaneous binding and unbinding of mGFP-labeled SARS-CoV-2 nucleocapsid proteins and cGAS-Halo^646^ to a single DNA molecule were monitored in real time using optical tweezers. Experiments were performed in a buffer containing 50 mM NaCl, 20 mM HEPES, pH 7.0, 5% glycerol, 2 mM DTT, and 0.3 mg/mL BSA. (*A* and *B*) Schematic of the optical tweezer microfluidic setup. Biotinylated λ-phage DNA was tethered between two optically trapped streptavidin-functionalized beads, then sequentially moved through four channels: (step ➊) 100 nM mGFP-N or mGFP-pN; (step ➋) 100 nM nucleocapsid protein and 10 nM cGAS–Halo^646^; (step ➌) nucleocapsid protein alone (100 nM); and (step ❹) plain buffer. Confocal line scans were used to generate kymographs across the DNA (48,503 bp) to visualize the DNA-binding kinetics of the proteins. (*C* and *D*) Representative kymographs showing mGFP-pN (*C*) or mGFP-N (*D*) binding to immobilized DNA and competition with cGAS–Halo^646^. For step ➋, pixel intensities are plotted as mean ± SD. Gray traces indicate cGAS–Halo^646^ intensities in the absence of nucleocapsid proteins (from Fig. 4*B*). While cGAS-Halo^646^ induces rapid mGFP-pN dissociation, prebound mGFP-N prevents stable cGAS-Halo^646^ binding to DNA.

For competition assays, we used nucleocapsid proteins in excess at a 10:1 stoichiometry, reflecting the rapid accumulation of SARS-CoV-2 nucleocapsid protein inside infected cells (19–21), while cGAS concentrations have been reported in the nanomolar range (9, 43). Using a microfluidic chip, we first equilibrated the λ-DNA with 100 nM mGFP-N or mGFP-pN protein (Fig. 5*A*). The DNA was then exposed to a mixture of 10 nM cGAS-Halo^646^ and 100 nM mGFP-N or mGFP-pN protein, followed by another incubation with the respective nucleocapsid protein alone (Fig. 5 *A* and *B*). Finally, the DNA was incubated in the buffer.

cGAS readily displaced prebound pN protein and remained stably bound to DNA in the buffer channel, indicating that pN protein does not interfere with DNA-cGAS interactions, consistent with our previous observations (Fig. 5*C*). In the presence of N protein, however, cGAS failed to establish stable interactions with DNA (Fig. 5*D*), supporting a competitive DNA-binding mechanism in line with our results using shorter DNA fragments (Fig. 1*G* and *SI Appendix*, Figs. S3 and S4). Notably, both nucleocapsid proteins exhibited dynamic interactions with DNA and rapidly dissociated from DNA upon tether transfer to the buffer channel, consistent with their mobility within condensates measured by FRAP (Fig. 3 *D* and *E*). In summary, we have directly visualized the competitive binding of the SARS-CoV-2 nucleocapsid protein to long DNA, which restricts cGAS binding and activation.

## Discussion

Our findings support a functional role for the SARS-CoV-2 nucleocapsid protein in suppressing cGAS-mediated immune responses triggered by cytoplasmic DNA in infected cells. We show that the N protein inhibits cGAS activation by binding to double-stranded DNA, thereby blocking cGAS interactions with its cognate DNA ligand. This competitive interaction occurs at nanomolar protein concentrations and extends to conditions where the nucleocapsid protein forms biomolecular condensates. These findings provide mechanistic insight into how SARS-CoV-2 evades host DNA-sensing pathways, complementing its established antagonism of RNA-sensing pathways.

Our observation that the nucleocapsid protein interacts with DNA aligns with its previously described promiscuous nucleic acid–binding behavior (44–46). Moreover, we show that the N protein binds double-stranded DNA with unexpectedly high affinity, effectively outcompeting cGAS for access to both short and long DNA molecules. This binding behavior suggests a potential role in suppressing cGAS activation by cytosolic DNA that may arise during SARS-CoV-2 infection. In addition to competing for DNA binding sites, the N protein may induce deformations in DNA to restrict cGAS engagement, consistent with the known sensitivity of cGAS for protein-induced alterations in DNA conformation (38, 40, 41, 47, 48).

At high concentrations, N protein sequesters DNA into biomolecular condensates (Fig. 3 *B* and *C* and *SI Appendix*, Figs. S5*C* and S6). Within these condensates, N protein–DNA interactions further prevent productive cGAS–DNA binding. Although cGAS can partition into these DNA-containing condensates, it remains inactive (Fig. 3 *B* and *C*)—a paradox given the highly dynamic nature of N–DNA interactions (Figs. 3*E* and 5*D*). This suggests that, while cGAS may be physically present within these condensates, it cannot interact with DNA in the specific conformation required for allosteric activation, and/or its interactions with DNA are too transient potentially preventing clustering to support stable binding and activity (Fig. 3*E*).

SARS-CoV-2 rewires cell signaling pathways and kinase activities upon infection, affecting the phosphorylation of both host and viral proteins. Phosphorylation of the SARS-CoV-2 nucleocapsid protein is detectable at several sites within 8 h after infection in cell culture (35). Host protein kinase families (SRPK, GSK-3, and CK1) have been shown to coordinately phosphorylate the serine/arginine-rich linker region of the nucleocapsid protein, which is required for viral replication (49). Members of the PKC superfamily, TTBK1/2, and EEF2K, as well as receptor tyrosine kinases EGFR and FGFR4, may also contribute to phosphorylation, as they have demonstrated the ability to phosphorylate the SARS-CoV-2 nucleocapsid protein at least in vitro (50). While several phosphoproteomic studies report phosphorylated sites of the SARS-CoV-2 nucleocapsid upon infection (35, 49, 50), the dynamics of the precise phosphorylation patterns and the fraction of the unphosphorylated protein during infection in humans remain unknown.

Extensive phosphorylation of the SARS-CoV-2 nucleocapsid protein has opposing effects during the viral lifecycle: While a high level of phosphorylation enhances viral genome replication, it impedes viral particle assembly (24, 51). Consequently, SARS-CoV-2 has to balance these conflicting roles of nucleocapsid protein phosphorylation (51). Mutations in circulating variants likely affect nucleocapsid protein phosphorylation differently (51) and might contribute to viral fitness differences observed. In this study, we add nucleic acid–sensing antagonism as yet another function affected by the phosphorylation state. It remains to be seen how nucleocapsid protein mutations of virus variants contribute to immune evasion by competitive DNA binding.

Our findings provide further evidence that phosphorylation serves as a molecular switch for nucleic acid binding by the nucleocapsid protein and extend this concept specifically to DNA binding (24, 27, 52). In its unphosphorylated state, the N protein binds to DNA with high affinity, effectively outcompeting cGAS, whereas the hyperphosphorylated nucleocapsid protein has no detectable effect on cGAS activation. This is consistent with previous work demonstrating that phosphorylation modulates the nucleocapsid protein’s propensity to self-associate and interact with nucleic acids (23–26, 36, 51, 52), and underscores its role in regulating nucleic acid–binding specificity during distinct stages of the viral life cycle.

Building on prior work by Cai et al. (16), which demonstrated that SARS-CoV-2 infection promotes mitochondrial DNA leakage and suppresses cGAS–STING signaling, our study presents a distinct mechanistic perspective. While Cai et al. proposed an indirect mechanism involving the modulation of G3BP1–cGAS interactions by the nucleocapsid protein and ruled out direct inhibitory effects on cGAS activity, we provide evidence for a direct competitive DNA-binding mechanism. We attribute this discrepancy to differences in protein purification strategies and assayed concentrations: Cai et al. employed a narrow range of roughly equimolar concentrations of N protein and cGAS (5 to 10 μM each) in their in vitro assays, under which we also observed no inhibition of cGAS activity. However, in infected cells, where the N protein is the first detectable viral protein and rapidly becomes one of the most abundant proteins (19, 20, 53), its concentration is expected to far exceed that of cGAS, enabling competitive binding. Additional differences between our studies result from the distinct protein purification strategies and experimental assays used (*SI Appendix*, *Materials and Methods*). Our findings suggest that protein stoichiometry plays a crucial role in cGAS inhibition, providing a potential model for how SARS-CoV-2 may suppress DNA sensing in infected cells.

A potential limitation of our study is the use of cell-free, in vitro reconstitution systems. While these approaches allow for precise dissection of the biophysical principles underlying nucleocapsid–DNA interactions, they lack the complexity of the cellular environment, which may impose additional constraints or regulatory factors. Nevertheless, our in vitro findings are consistent with cellular studies, which have observed the immunosuppressive effect of the N protein in both SARS-CoV-2 infection and N protein overexpression models, further supporting the physiological relevance of our conclusions. Indeed, during infection, proinflammatory interferon responses appear to be primarily generated by noninfected bystander cells, rather than by infected cells themselves (18, 54, 55).

In conclusion, our study uncovers a direct inhibitory mechanism by which the SARS-CoV-2 nucleocapsid protein competitively binds to cytosolic DNA, thereby preventing cGAS activation. This immune evasion strategy may represent a crucial component of the virus’s ability to suppress interferon responses during the early stages of infection. This immune antagonism may be critical for viral fitness, as SARS-CoV-2 replication is sensitive to cGAS–STING signaling, with tonic signaling restricting viral replication (54, 55). The DNA-targeting mechanism described here complements the N protein’s known role in antagonizing RNA-sensing pathways (3), underscoring its multifaceted role in immune evasion. While this study focused on cGAS, the N protein may similarly interfere with other DNA sensors, such as AIM2 or IFI16. Our findings raise the possibility that nucleocapsid proteins from other RNA viruses may similarly subvert DNA-sensing pathways, revealing an additional layer of complexity in virus–host interactions. Targeting these nucleocapsid protein–DNA interactions may represent a potential strategy for antiviral therapeutic development.

## Materials and Methods

cGAS and nucleocapsid protein variants were expressed in suspension-adapted insect cells of *Trichoplusia ni* (Tni) and *Spodoptera frugiperda* (Sf9) using baculovirus infection, leveraging on the FlexiBAC system (56). Proteins were purified using at least three-step chromatographic procedures to remove nucleic acid and protein contaminants and were subjected to quality control. Details on the plasmid constructs and purified proteins used in the experiments are provided in *SI Appendix*, Table S1. The oligonucleotide sequences used in this study are provided in *SI Appendix*, Table S2. The protein expression and purification procedures, including site-specific fluorescent labeling and biotinylation, are detailed in *SI Appendix*, *Materials and Methods*.

Materials, methods, and data analysis for polyacrylamide gel electrophoresis, analytical size-exclusion chromatography coupled to static light scattering, mass spectrometry, mass photometry, nano-differentialscanning fluorimetry (nanoDSF), in vitro cGAMP production and detection by thin-layer chromatography, microscale thermophoresis, biolayer interferometry, microscopy-based phase separation assays, FRAP, and optical tweezers coupled to confocal microscopy experiments are described in *SI Appendix*, *Materials and Methods*.

## Supplementary Material

Appendix 01 (PDF)

## Data Availability

Source data have been deposited in Zenodo (https://zenodo.org/records/14475043) (57). Data and materials are also available from the corresponding authors upon reasonable request.
