# Supplementary material for: SARS-CoV-2 nucleocapsid protein directly prevents cGAS–DNA recognition through competitive binding
Source: Proc Natl Acad Sci U S A. 2025 Jun 23;122(26):e2426204122. doi: 10.1073/pnas.2426204122 (PMC12232725; doi:10.1073/pnas.2426204122)
Supplement: Supplementary file 1 — Appendix 01 (PDF) [file pnas.2426204122.sapp.pdf]

PNAS

Supporting Information for

SARS-CoV-2 nucleocapsid protein directly prevents cGAS–DNA recognition through competitive binding

Theresia Gutmann[a,1,2]✉, David Kuster[a,1], Anthony A. Hyman[a,1,2]✉

[a] Max Planck Institute of Cell Biology and Genetics, 01307 Dresden, Germany

[1] T.G. and D.K. contributed equally to this work.

[2] To whom correspondence may be addressed.
Email: ✉ gutmann@mpi-cbg.de or ✉ hyman@mpi-cbg.de.

This PDF file includes:

Supporting text
Figures S1 to S6
Tables S1 to S2
SI References

Table of Contents

Table of Contents 2
Supporting Information Text 3
Materials and Methods 3
Cell culture and baculovirus production 3
Protein expression and purification 3
Site-specific cGAS-Halo labeling 5
Site-specific biotinylation of AviTag-cGAS^{CD} 5
Analytical size exclusion chromatography coupled to static light scattering 6
Polyacrylamide electrophoresis 6
Mass photometry 7
Thermal stability assessment by nano-differential scanning fluorimetry 7
In vitro cGAS activity assay and cGAMP detection 8
Microscale thermophoresis experiments 8
Bio-layer interferometry 8
Microscopy-based phase separation assays 9
Fluorescence recovery after photobleaching (FRAP) 10
Optical tweezers coupled to confocal microscopy 10
Data analysis and visualization 11
Data availability 11
Fig. S1. Purification of full-length human cGAS from insect cells. 12
Fig. S2. Purification of full-length SARS-CoV-2 N and pN proteins. 13
Fig. S3. Excess DNA reverses N protein inhibition of cGAS activity 15
Fig. S4: The SARS-CoV-2 N protein accelerates DNA dissociation from cGAS. 15
Fig. S5: cGAS partitioning in nucleocapsid protein condensates. 16
Fig. S6: Cy5 labeling does not affect DNA partitioning or urea sensitivity of DNA–protein complexes. 18
Table S1. Recombinant proteins and plasmids used in this study. 19
Table S2. Oligonucleotides used in this study. 20
SI References 21

Supporting Information Text

Materials and Methods

Cell culture and baculovirus production

Suspension-adapted Spodoptera frugiperda (Sf9) cells (RRID:CVCL_0549, Expression Systems, Davis, CA, USA, 94-001F) and Trichoplusia ni (Tni) cells (RRID:CVCL_C412, Expression Systems, 94-002) were cultured in protein-free ESF921 insect cell medium (Expression Systems, 96-001-01) supplemented with 1% Penicillin-Streptomycin (Gibco, 15140122) in glass culture flasks at 27 °C and 100 rpm. Recombinant baculoviruses were produced in Sf9 cells using the engineered FlexiBAC system as described (1).

Protein expression and purification

Synthetic codon-optimized genes, flanked by NotI and AscI restriction sites, were synthesized by Twist Bioscience (South San Francisco, CA, USA) or GenScript (Piscataway, NJ, USA) and were inserted into the respective pOCC shuttle vectors leveraging the FlexiBAC system (1) by restriction cloning for baculovirus production. All expression constructs contained a cleavable N-terminal maltose-binding protein (MBP) tag followed by a flexible linker sequence (NSSSNNNNNNNNNNSSGR) and a human rhinovirus 3C protease cleavage site. Protein extinction coefficients and isoelectric points were predicted using the ExPASy tool (2). The list of recombinant proteins and respective plasmids is provided in Table S1.

cGAS cloning and purification

The gene sequence encoding full-length human cGAS (UniProt ID: Q8N884-1, 1-522) or truncated $cGAS^{CD}$ (UniProt ID: Q8N884-1, 158-522) was codon-optimized for human expression, flanked by NotI and AscI restriction sites and cloned into appropriate pOCC shuttle vectors compatible with the FlexiBAC baculovirus system (1). Full-length cGAS, mGFP-cGAS, cGAS-Halo, and AviTag-$cGAS^{CD}$ were expressed in baculovirus-infected Tni cells as MBP-tagged fusion proteins and purified as described below for His_6-MBP-3C-cGAS.

His_6-MBP-3C-cGAS was expressed in suspension-adapted Tni cells by infecting a 500 mL culture at a density of 1×10^6 cells/mL with 5 mL of P2 baculovirus stock. Cells were incubated at 27 °C and 100 rpm for 63 hours before harvesting by centrifugation (300 × g, 10 min, room temperature). Cell pellets were resuspended in lysis buffer (500 mM NaCl, 50 mM HEPES, pH 7.5, 2 mM $MgCl_2$, 2 mM DTT, 10% glycerol, 1× EDTA-free protease inhibitors (Roche, 05056489001), and 0.25 U/mL Benzonase (produced in-house)). Cells were lysed on ice by sonication using a Branson Digital Sonifier 450 (Branson Ultrasonics; 2 cycles of 90 s, 30% amplitude, 3 s pulse on, 7 s pulse off). The lysate was cleared by centrifugation (Type 45 Ti rotor; 20,000 rpm; 1 h; 4 °C).

The supernatant was filtered through a 0.45 µm bottle-top filter (Sarstedt, 83.3941.100) and incubated with 12 mL amylose resin (New England Biolabs, E8021L) for 30 min at 4 °C in batch. The bead-lysate slurry was loaded onto a filtration column with double polyethylene frits (Thermo Fisher Scientific, 12946089). The beads were washed with 2.5 column volumes (CV) of wash buffer I (500 mM NaCl, 50 mM HEPES, pH 7.5, 10% glycerol, 1 mM DTT) followed by 2.5 CV of wash buffer II (1 M NaCl, 50 mM HEPES, pH 7.5, 1 mM DTT), and 8 CV of wash buffer III (500 mM NaCl, 50 mM HEPES, pH 7.5, 1 mM DTT). His_6-MBP-cGAS was eluted with 500 mM NaCl,

50 mM HEPES, pH 7.5, 1 mM DTT, and 10 mM maltose. The MBP purification tag was cleaved with GST-tagged HRV 3C protease (produced in-house) for 2 h at 4 °C (1:100 w/w).

To remove nucleic acid or protein contaminants, the protein was further purified by heparin affinity chromatography. The eluate was diluted to an ionic strength of ~160 mM NaCl, filtered (Millipore, S2GPT01RE), and loaded onto a 5 mL HiTrap Heparin HP column (Cytiva, 17040703) at a flow rate of 5 mL/min. The column was washed with 15 CV 150 mM NaCl, 20 mM HEPES, pH 7.5, followed by elution in a 150–1000 mM NaCl gradient over 15 CV. The cGAS-containing peak fractions were concentrated to ~12 mg/mL using Amicon Ultra-15 centrifugal filters (30 kDa MWCO, Millipore, UFC903024) and subjected to size-exclusion chromatography using a Superdex 200 Increase 10/300 GL column (Cytiva, 28990944) in 500 mM KCl, 20 mM HEPES, pH 7.5, 0.5 mM TCEP, and a flow rate of 0.5 mL/min. Peak fractions were pooled, concentrated to ~10 mg/mL (Millipore, UFC803024), aliquoted, and flash-frozen for storage at -80 °C. Protein concentration was determined by UV absorbance at 280 nm, and the 260 nm/280 nm absorbance ratios were monitored (~0.59). cGAS-Halo, mGFP-cGAS, and AviTag-cGAS^{CD} were purified analogously.

SARS-CoV-2 nucleocapsid protein cloning and purification

The nucleocapsid protein coding sequence from the SARS-CoV-2 isolate Wuhan-Hu-1 (GenBank: MN908947.3, positions +28,274 to +29,533; Uniprot identifier P0DTC9) (3) was codon-optimized for insect cell expression, flanked by NotI and AscI restriction sites, and cloned into pOCC shuttle vectors using restriction cloning for baculovirus production (1). SARS-CoV-2 nucleocapsid proteins were purified as described (4).

A 500 mL Sf9 cell culture at a density of ~10^6 cells/mL was infected with 2% v/v baculoviral supernatant and subsequently grown for 72 h at 27 °C and 100 rpm. Cells were harvested by centrifugation at 1,000 × g for 5 min, and cell pellets were flash-frozen in liquid nitrogen and stored at -80 °C. The cells were lysed in 50 mL cold buffer (1 M NaCl, 50 mM $Na_xH_xPO_4$, 20 mM imidazole, 5% v/v glycerol, 4 mM $MgCl_2$, 1 mM DTT, 1× EDTA-free protease inhibitors (Roche), 4 U/mL DNase I (Roche, 04716728001), pH 7.4) using an LM20 microfluidizer (Microfluidics, Westwood, MA, USA) at 15,000 psi. The lysate was cleared by ultracentrifugation (Type 45 Ti rotor; 30,000 rpm; 30 min; 4 °C) and passed through a 0.45 µm filter (Corning, 430514) followed by a three-step liquid chromatography purification process at room temperature.

The protein was first purified by affinity chromatography using a 5 mL HisTrap FF column (Cytiva, 17-5255-01) at a flow rate of 5 mL/min. After loading, the column was washed with 8 CV imidazole wash buffer (150 mM NaCl, 50 mM $Na_xH_xPO_4$, 20 mM imidazole, 5% v/v glycerol, pH 7.4), and the protein was eluted in a 40‑300 mM imidazole gradient over 10 CV. Fractions containing N protein were pooled and supplemented with an additional 400 mM NaCl, 150 mM Arg-HCl (pH 7.4), and 300 mM trehalose to prevent aggregation during volume reduction in Amicon Ultra-15 centrifugal filters (30 kDa MWCO, Millipore, UFC903024) to ~5 mL. The solubility and purification tags were cleaved with 500 µg HRV-3C protease-His_6 (produced in-house) in the presence of 0.5 mM DTT for 1 h at 25 °C.

Upon dilution to a final NaCl concentration of ~150 mM, the proteins were passed through a 5 mL HiTrap Heparin HP column (Cytiva), followed by a wash step with 8 CV heparin wash buffer (150 mM NaCl, 50 mM $Na_xH_xPO_4$, 5% v/v glycerol, pH 7.4) and a 150‑1000 mM NaCl gradient elution over 10 CV. Peak fractions containing nucleocapsid protein were pooled, supplemented

with an additional 700 mM NaCl, 150 mM Arg-HCl (pH 7.4), and 300 mM trehalose to prevent aggregation, and concentrated in an Amicon Ultra-15 centrifugal filter (30 kDa MWCO) to ~2 mL.

The eluate was split, with one portion directly subjected to size-exclusion chromatography to purify the hyperphosphorylated nucleocapsid (pN) protein. The other portion was incubated with 500 µg λ-phosphatase in reaction buffer (500 mM NaCl, 25 mM $Na_xH_xPO_4$, pH 7.4, 2.5% v/v glycerol) for 1 h at 30 °C, and then subjected to size-exclusion chromatography to purify the dephosphorylated nucleocapsid (N) protein.

The samples were passed through a 0.2 µm spin filter (Agilent Technologies, 5185-5990) and resolved by size-exclusion chromatography on a Superdex 200 Increase 10/300 GL column in 50 mM $Na_xH_xPO_4$, 300 mM NaCl, 5% v/v glycerol, 1 mM DTT, pH 7.4 at a flow rate of 0.5 mL/min. Peak fractions were pooled and concentrated to ~200 µL using Amicon Ultra-4 centrifugal filters (30 kDa MWCO). The protein concentration was determined at 280 nm, and 260 nm/280 nm absorbance ratios below ~0.58 indicated the absence of nucleic acid contaminants. Aliquots of 5 µL were prepared, flash-frozen in liquid nitrogen, and stored at -80 °C.

Intact protein mass spectrometry

Intact-mass measurements were performed by Spectroswiss (Lausanne, Switzerland) to determine the mass of protein monomers and assess the degree of phosphorylation, using a Q Exactive HF BioPharma Orbitrap Fourier transform mass spectrometry (FTMS) system. Data were processed using proprietary data processing workflows (Peak-by-Peak BioPharma, Spectroswiss).

15 µg of pN protein was subjected to SEC-MS under native conditions. For the pN protein, several proteoforms were detected, with the most abundant having a mass of 47,417.1 Da, consistent with up to 15 phosphoryl groups (Fig. S2I). Other proteoforms may contain 13 and 14 phosphoryl groups.

The intact mass of the N protein was mass was determined under denaturing conditions using 0.2 µg of reversed-phase HPLC-MS. Complete dephosphorylation was confirmed with an observed molecular mass of 46,217.2 Da (theoretical mass of the unmodified N protein: 46,217.8 Da) (Fig. S2I).

Site-specific cGAS-Halo labeling

Recombinant cGAS-Halo was labeled with the Janelia Fluor 646 HaloTag Ligand (646) (Promega, GA1120) for 18 h at 4 °C, and free dye was removed by size-exclusion chromatography on a Superdex 200 Increase 10/300 GL column with 250 mM KCl, 20 mM HEPES, pH 7.5, 0.5 mM TCEP as the running buffer. The peak fraction containing cGAS-Halo^{646} was concentrated to 15 µM and flash-frozen in single-use aliquots. Labeling efficiency was ~33%.

Site-specific biotinylation of AviTag-cGAS^{CD}

Biotinylation of AviTag-cGAS^{CD} was carried out as described in (5). Briefly, 56 µM AviTag-cGAS^{CD} (187 nmol) was incubated with 68 µM (~225 nmol) biotin, 5.6 µM GST-tagged *E. coli* biotin ligase (BirA) in 110 mM KCl, 20 mM HEPES, pH 7.5, 10 mM $MgCl_2$, 5 mM ATP, and 0.25 mM TCEP in a total volume of 3.3 mL at 4 °C for 18 h. BirA-GST was subsequently removed by reverse GST-affinity chromatography using 300 µL Glutathione Sepharose 4 B resin (Cytiva, 17075601). The sample was concentrated using Amicon Ultra-4 centrifugal filters (30 kDa MWCO), and free biotin

was removed by size-exclusion chromatography on a Superdex 200 Increase 10/300 GL column equilibrated with 20 mM HEPES, pH 7.5, 300 mM NaCl, 5% glycerol, and 0.5 mM TCEP at a flow rate of 0.45 mL/min at 8 °C. Peak fractions containing the monomeric biotinylated AviTag-cGAS^{CD} were pooled, diluted to a final concentration of 10 µM, flash-frozen in single-use aliquots in liquid nitrogen, and stored at -80 °C. A gel shift assay confirmed a biotinylation efficiency of greater than 95%. The conformational thermostability of the biotinylated protein was confirmed by nanoDSF in 300 mM NaCl, 20 mM HEPES, pH 7.5, 0.5 mM TCEP, 5% glycerol (melting temperatures: AviTag-cGAS^{CD} 47.9 ± 0.0 °C; biotin-AviTag-cGAS^{CD} 47.3 ± 0.1 °C).

Analytical size exclusion chromatography coupled to static light scattering

Analytical size exclusion chromatography (SEC) coupled to static light scattering (SLS) was performed using a Superdex 200 Increase 10/300 GL column. Proteins were diluted to 1-3 mg/mL in the respective SEC buffer, filtered through 0.22 µm spin filters (Costar, 8161), and 100 µL of each sample was analyzed on the equilibrated column at a flow rate of 0.5 mL/min at room temperature. The SEC buffer was 500 mM KCl, 20 mM HEPES, pH 7.5, 0.5 mM TCEP (for cGAS proteins) or 300 mM NaCl, 50 mM $Na_xH_xPO_4$, pH 7.4, 5% v/v glycerol, 1 mM DTT (for nucleocapsid proteins). Proteins were characterized by SEC and dual-angle light scattering detection at 7° and 90° using a Viscotek VE 2001 GPCmax (Malvern Pananalytical, Malvern, UK) and a Viscotek TDA 305 detection system (Malvern Pananalytical). Calibration was performed with bovine serum albumin (BSA, Sigma, 7638-5g) standards. The data were analyzed using the OmniSEC 4.7.0 software.

Polyacrylamide electrophoresis

Proteins were separated by denaturing sodium dodecyl sulfate-polyacrylamide gel electrophoresis (SDS-PAGE) using reducing Laemmli SDS sample buffer and 4–12% Bis-Tris gels (Invitrogen, NP0322BOX) with 1× MOPS SDS running buffer (Invitrogen, NP000102) at 190 V for 50 min.

To assess phosphorylation, denaturing Phos-tag gel electrophoresis was employed, which relies on characteristic retardation of protein species within the gel matrix copolymerized with a Zn^{2+}-chelating Phos-tag moiety. Phos-tag gel electrophoresis was performed using non-reducing Laemmli SDS sample buffer and precast 7.5% SuperSep Phos-tag gels (FujiFilm Wako, 192-18001) with 1× Tris-Glycine SDS running buffer at 150 V for 2 h. Molecular weight markers used were SeeBlue Pre-stained Protein Standard (Invitrogen, LC5625) or PageRuler Plus Prestained Protein Ladder 10–250 kDa (Thermo Fisher Scientific, 26619).

Polyacrylamide gels were stained with Coomassie Brilliant Blue R-250 (SERVA Electrophoresis, 17525) and destained in 10% ethanol and 5% acetic acid in H_2O (for cGAS proteins) or stained with InstantBlue protein stain (Sigma-Aldrich, ISB1L) and destained in 1 M sodium chloride (for nucleocapsid proteins).

Denaturing urea-PAGE

To assess the impact of Cy5 labeling on DNA partitioning and protein binding, condensates were formed using increasing amounts of either Cy5-labeled or label-free 50-bp DNA oligonucleotides, followed by the quantification of apparent free DNA in the dilute phase. Reactions were assembled in PCR tubes in a final volume of 18 µL, containing 10 µM N protein, 10 µM pN protein, or 1 µM

cGAS, in the presence of 0.25, 0.5, or 1 µM 50-bp oligonucleotides, in low-salt buffer (50 mM NaCl, 20 mM HEPES, pH 7.5, 5% glycerol, 2 mM DTT, 5 mM $MgCl_2$, 0.1 mM ATP, and 0.1 mM GTP). Samples were equilibrated at room temperature for 2 h. To separate the condensed and dilute phases, samples were centrifuged at 20,000 × g for 15 min at 23 °C. The top 10 µL of supernatant (dilute phase) was carefully removed and mixed with 10 µL of 2× urea buffer (8 M urea, 50 mM EDTA, bromophenol blue), followed by heat denaturation at 70 °C for 3 min. Samples were separated by denaturing electrophoresis using 10% TBE–Urea polyacrylamide gels (Invitrogen, EC6875BOX) and 1× TBE buffer (Invitrogen, LC6675).

Gels were imaged for Cy5 fluorescence using a Typhoon FLA 9500 laser scanner (Cytiva) with a 635-nm laser. To detect DNA, gels were stained with 1 µg/mL ethidium bromide for 20 min, destained in H_2O, and imaged under ultraviolet light with an F590 filter on a gel-documentation system (Vilber-Lourmat, BIO-VISION-3026 WL/LC/26LMX). A 25-bp DNA ladder (Applied Biosystems, 931343) was used as a marker.

Apparent free DNA was analyzed by densitometry using Fiji (6) (Fig. S6B). Signal intensities were first normalized to the protein-free 50-bp DNA-Cy5 control at the highest concentration (1 µM) (Fig. S6B). The apparent free DNA fraction was then determined by dividing the signal intensity of each sample by its corresponding protein-free DNA control (Fig. S6C). No apparent difference in partitioning or binding behavior was observed between Cy5-labeled and label-free DNA across all conditions.

Mass photometry

Mass photometry experiments were performed using a TwoMP instrument (Refeyn, UK, Oxford). High-precision coverslips (24 × 50 mm, thickness 1.5H (170 µm ± 5 µm); Carl Roth, LH25.2) were cleaned by sonication for 5 min in isopropanol, followed by two rinses with milli-Q H_2O, sonicated again for 5 min in milli-Q H_2O, and dried using nitrogen gas. A 3 × 2 well silicone gasket (Grace Bio-Labs, 103250) was placed at the center of a coverslip, which was then positioned on the microscope stage. 18 µL of sample buffer (150 mM KCl, 20 mM HEPES, pH 7.5, 1 mM DTT) was added to a well to focus the objective. Next, 2 µL of the recombinant protein solution (prediluted in sample buffer to 100 nM) was added to the buffer, resulting in a final protein concentration of 10 nM. The solution was mixed by pipetting and measured immediately. The mass photometer was calibrated using BSA (Sigma, I4506) and human immunoglobulin (Thermo Fisher Scientific, 23209) standards. Mass photometry data were acquired using Acquire^{MP} (Refeyn, version 2.5.0), and mass distributions were analyzed using the Discover^{MP} software (Refeyn, version 2.5.0). Representative data are presented as a histogram of individual mass measurements, with peaks fitted using a Gaussian function to determine the mean molecular mass (μ) and variability (standard deviation, σ):

$$f(x) = a \cdot \mathrm{e}^{\left[-\frac{(x-\mu)^2}{2\,\sigma^2}\right]}$$

where $f(x)$ is the frequency of particles at mass x, and a is the amplitude of the peak.

Thermal stability assessment by nano-differential scanning fluorimetry

The thermal stability of the recombinant proteins was assessed by nano-differential scanning fluorimetry (nanoDSF). Protein samples were diluted to a concentration of 5 µM in their respective protein storage buffers, loaded into nanoDSF Grade Standard capillaries (PR-C002,

NanoTemper Technologies, Munich, Germany), and analyzed on a Prometheus NT.48 instrument (NanoTemper Technologies). Thermal unfolding was detected by recording the intrinsic tryptophan fluorescence shift (emission ratio at 350 and 330 nm) during heating in a linear thermal ramp (20 °C to 95 °C) with a rate of 1 °C/min (cGAS proteins) or 0.1 °C/min (nucleocapsid proteins) with an excitation power of 40%. Data were processed using the NanoTemper PR.ThermControl software (version 2.1.1) and exported as.csv files.

In vitro cGAS activity assay and cGAMP detection

The enzymatic reaction was carried out using 1 µM recombinant cGAS and 0.20 µM dsDNA-100 (~1.25 µM binding sites) in a buffer containing 120 mM Na/KCl, 20 mM HEPES, pH 7.5, 5 mM $MgCl_2$, 0.5 mM ATP, 0.5 mM GTP, 1 mM DTT with varying concentrations of nucleocapsid protein for 3 h at room temperature, if not indicated otherwise. The reaction was stopped by heating to 95 °C for 3 min. cGAMP production was detected by thin-layer chromatography. 3-µL samples were spotted onto a 10 × 10 cm silica gel high-performance thin-layer chromatography (HPTLC) plate containing a fluorescent indicator F_{254} (Merck, 105628), and separation was performed in an *n*-propanol/ammonium hydroxide/water (11:7:2 [vol/vol/vol]) solvent system (7) for ~90 min. The plate was air-dried, and bands were visualized with ultraviolet light at 254 nm. Densitometry analysis was performed in Fiji after background subtraction using the rolling ball algorithm (radius: 50 pixels).

Microscale thermophoresis experiments

Microscale thermophoresis experiments were carried out using Cyanin 5 (Cy5)-labeled oligonucleotides, either 50-bp DNA-Cy5 or 50-nt RNA-Cy5, at a concentration of 0.75 nM in 150 mM NaCl, 20 mM HEPES, pH 7.5, 1 mM DTT, and 0.05% Tween-20. Serial dilutions of nucleocapsid proteins were first prepared in a buffer containing 300 mM NaCl, 20 mM HEPES, pH 7.5, 1 mM DTT, and 0.05% Tween-20 to ensure sample homogeneity. Cy5-labeled oligos were added 1:1 to each sample in 75 mM NaCl, 20 mM HEPES, pH 7.5, 1 mM DTT, and 0.05% Tween-20 to achieve a final oligo concentration of 0.75 nM. The samples were incubated for 30 minutes at room temperature before analysis in premium capillaries (MO-K025, NanoTemper Technologies) on a Monolith NT.115 Pico instrument (NanoTemper Technologies) using the Pico-RED detector with 30% light-emitting diode power at 25 °C. The integral of thermophoresis traces from 4 to 5 s on-time was used for binding affinity determination, and the normalized fluorescence difference ΔF_{norm} was plotted against ligand concentration for dose-response plots. To determine K_d values, nonlinear regression was performed with Python and the Hill equation:

$$f = b + \frac{b - m}{1 + \left(\frac{K_d}{c_i}\right)^{n_H}}$$

where c_i is the molar protein concentration, K_d is the apparent dissociation constant, n_H is the Hill coefficient reflecting cooperativity, and m and b are the normalization factors for the lower and upper asymptotes of the titration curve, respectively.

Bio-layer interferometry

Bio-layer interferometry (BLI) measurements were carried out at 25 °C using a GatorPlus system (GatorBio, Palo Alto, CA, USA) controlled by GatorOne Software (version 2.15.5.1221). The shaker speed was set to 1,000 rpm, and data were acquired at 5 Hz. Reactions were performed in BLI

buffer (150 mM KCl, 20 mM HEPES, pH 7.5, 0.05% (v/v) Tween-20). Site-specifically biotinylated AviTag-cGAS^{CD} (100 nM) was immobilized onto streptavidin probes (GatorBio, 160002) for ~140 s. The biosensors were washed in BLI buffer for 60 seconds and then transferred to wells containing 20-bp DNA or 45-bp DNA in BLI buffer for 120 seconds to saturate the immobilized cGAS with DNA. The biosensors were then dipped into wells containing various concentrations of the N protein (0-250 nM) in BLI buffer, and binding and unbinding were monitored. Two independent experiments were performed for each sample.

Microscopy-based phase separation assays

Nucleocapsid protein condensates were prepared by diluting the recombinant proteins to 10 μM N or pN protein (containing 10 mol% fluorescently labeled nucleocapsid protein), with or without 0.5 μM 50-bp Cy-5-labeled DNA, and 1 μM mGFP-cGAS or cGAS-Halo^{646} in the final buffer composed of 50 mM NaCl, 20 mM HEPES, pH 7.5, 5 mM $MgCl_2$, 0.1 mM ATP, 0.1 mM GTP, 5% glycerol, and 2 mM DTT, unless stated otherwise. Samples were prepared in a total volume of 20 μL in black 384-well ultra-low attachment plates (Perkin Elmer, 6057302) and incubated for 45 min before imaging at room temperature.

Condensates were imaged at room temperature after equilibrium was reached using a Nikon Eclipse Ti inverted spinning disk confocal microscope equipped with a CSU-X1 scan head (Yokogawa, Musashino, Japan) and a 60 × 1.2 NA Plan Apochromat VC water-immersion objective (Nikon, Minato, Japan). Samples were illuminated with 488-nm, 561-nm, and 660-nm diode-pumped solid-state lasers, and images were captured using an iXON 897 EMCCD camera (Andor, Belfast, Northern Ireland). Images were randomly acquired within each well using an automated grid function within the acquisition software iQ (version 3.4.1; Andor).

Image processing was performed using Fiji (version 2.14.0) (6). Region-of-interest (ROIs) were defined by segmenting condensates in the nucleocapsid protein channel based on fluorescence intensity thresholding using an in-house Python script (provided by the Scientific Computing Facility at MPI-CBG). The resulting ROIs were applied to all image channels. Mean fluorescence intensity values for condensed and dilute phases were background-subtracted to correct for intrinsic camera noise using images acquired from a buffer-only sample. 49 fields-of-view were analyzed per condition. Ratios of fluorescence intensity between the condensed and dilute phases were calculated as an estimate of relative cGAS or nucleocapsid enrichment within the condensed phase.

The contribution of electrostatic interactions to homotypic nucleocapsid protein condensation was evaluated by assessing the dependence of condensate formation on ionic strength. Briefly, nucleocapsid protein (10 μM, containing 10 mol% GFP-tagged protein) was incubated for 1 h at room temperature in assay buffer (20 mM HEPES, pH 7.5, 5 mM $MgCl_2$) with NaCl concentrations ranging from 50 to 1000 mM in a total volume of 20 μL. Assays were carried out in black 384-well ultra-low attachment plates and imaged as detailed above. Briefly, 49 fields-of-view were acquired in z-stacks covering a height of 10 μm in 11 z-planes, using the automated grid function within the acquisition software iQ. Maximum intensity projections were performed for each field-of-view.

These were further analyzed in Fiji with an in-house Python script, as indicated above, for segmentation of condensates and dilute phase and fluorescence intensity measurements. This allowed the calculation of the 'relative droplet fraction' for a given field-of-view as previously

described (8), which was then fitted to the Hill equation. The threshold for complete condensate dissolution was arbitrarily defined as a relative droplet fraction of 0.05. The corresponding critical sodium chloride concentrations ($[NaCl]_{0.05}$) were derived from the intersection of the fitted curve and the threshold value.

Fluorescence recovery after photobleaching (FRAP)

FRAP experiments were conducted on nucleocapsid protein condensates formed as described above. Condensates were formed by diluting 10 µM nucleocapsid protein (containing 10 mol% mCherry-labeled fraction), 0.5 µM 50-bp DNA-Cy5, and 1 µM mGFP-cGAS in a buffer with the final composition of 50 mM NaCl, 20 mM HEPES, 5 mM $MgCl_2$, 0.1 mM ATP, 0.1 mM GTP, 5% glycerol, 2 mM DTT. FRAP measurements were carried out within 1–2 h after initiating droplet formation when steady-state conditions were reached. Upon acquisition of three prebleach frames, up to six individual condensates per field-of-view were fully photobleached within a 50 x 50 px^2 (~9 x 9 μm^2) area using the 488-nm laser at 100% laser power for 20 consecutive repeats with a 60-msec dwell time each. Fluorescence recovery was recorded using 561-nm (mCherry) and 488-nm (GFP) lasers at a rate of 0.5 fps for the first 40 s, then 0.33 fps for 60 s, and 0.2 fps for the remaining 500 s (in total, a 10-min acquisition time). To account for the movement of condensates relative to the objective during acquisition time, recursive alignment was performed over the stack of cropped images using Fiji: The image registration tool MultiStackReg was used in the 'rigid body' transformation mode, correcting for translation and rotation of condensates based on the 561-nm excitation channel (nucleocapsid protein signal). Condensates were manually segmented by defining a circular ROI with a 9 µm diameter. The background was subtracted for each time point, using ROIs in the dilute phase of a given field-of-view. Fluorescence intensities were normalized relative to fluorescence intensities of prebleached condensates, yielding a range between 0 and 1, and fitted to the exponential equation:

$$f(t) = A \cdot (1 - \mathrm{e}^{-\tau t})$$

where $f(t)$ is the fluorescence intensity at a time after bleaching t, A is the mobile fraction, and τ is the rate constant for the recovery. The half-time of fluorescence recovery $t_{1/2}$ was calculated as:

$$t_{1/2} = \frac{\ln 0.5}{-\tau}.$$

Optical tweezers coupled to confocal microscopy

Experiments were performed using a C-Trap optical tweezers system (first generation; Lumicks, Amsterdam, NL) equipped with dual optical traps, integrated confocal fluorescence microscopy, and a u-Flux microfluidics system. The system was configured with a Nikon CFI Plan Apo 60x water-immersion objective (1.2 NA) and a Leica P 1.40 NA oil-immersion condenser. The trapping laser was set to 100%, with an overall power of 30–35% relative to maximum output, resulting in a trap stiffness of ~0.33 pN/nm. A 488-nm laser (5% power, emission filter 525/40 nm) and a 638-nm laser (10% power, emission filter 680/42 nm) were used for confocal imaging. The dwell time per pixel was 0.05 ms, and the pixel size was 100 × 100 nm. Streptavidin-coated polystyrene beads (diameter 4.37 µm, Spherotech, SVP-40-5) were diluted to 0.002% w/v, and biotinylated double-stranded bacteriophage λ DNA (48,502-bp; Lumicks, SKU 00001) was diluted to 20 pg/µL in 50 mM NaCl, 20 mM HEPES, pH 7.0. Proteins were diluted in binding buffer (50 mM NaCl,

20 mM HEPES, pH 7.0, 5% glycerol, 2 mM DTT, and 0.3 mg/mL BSA) to the final concentrations indicated.

Experiments, including bead trapping, DNA immobilization, and imaging, were automated using custom Python scripts via the Bluelake API (version 2.4; Lumicks). A single biotinylated λ DNA molecule was tethered between two 4.37 μm streptavidin-coated polystyrene beads using laminar flow. The trap-to-trap distance was maintained at 16 μm for all binding and unbinding experiments. After immobilizing the DNA tether, the system was moved sequentially through microfluidic channels and imaged in each channel for 120 s at 2 fps. For cGAS binding and unbinding experiments (Fig. 4), the tether was imaged sequentially in: (1) a protein channel containing 10 nM cGAS-Halo^{646}, and (2) a channel containing plain binding buffer. For competition binding experiments (Fig. 5), the DNA tether was incubated in (1) protein channel 1 containing 100 nM mGFP-labeled nucleocapsid protein, (2) protein channel 2 containing 100 nM mGFP-labeled nucleocapsid protein and 10 nM cGAS-Halo^{646}, (3) protein channel 1 containing 100 nM mGFP-labeled nucleocapsid protein, and finally in (4) buffer channel containing plain binding buffer.

Data were exported and analyzed using the Python package PyLake (9). Kymograph projections were generated along the DNA tether to visualize the dynamics of nucleocapsid and cGAS binding. Each experimental condition was independently replicated at least three times.

Data analysis and visualization

Data were analyzed and visualized using Python (3.12.4) and the following Python libraries: pandas (version 2.2.2), NumPy (version 2.1.0), matplotlib (version 3.9.1), seaborn (version 0.13.2), SciPy (version 1.14.1), and lumicks.pylake (version 1.5.2) if not indicated otherwise. The structural model in Figure 1A was produced in UCSF ChimeraX (version 1.8) (10).

Data availability

The source data supporting this study have been deposited on Zenodo (11).

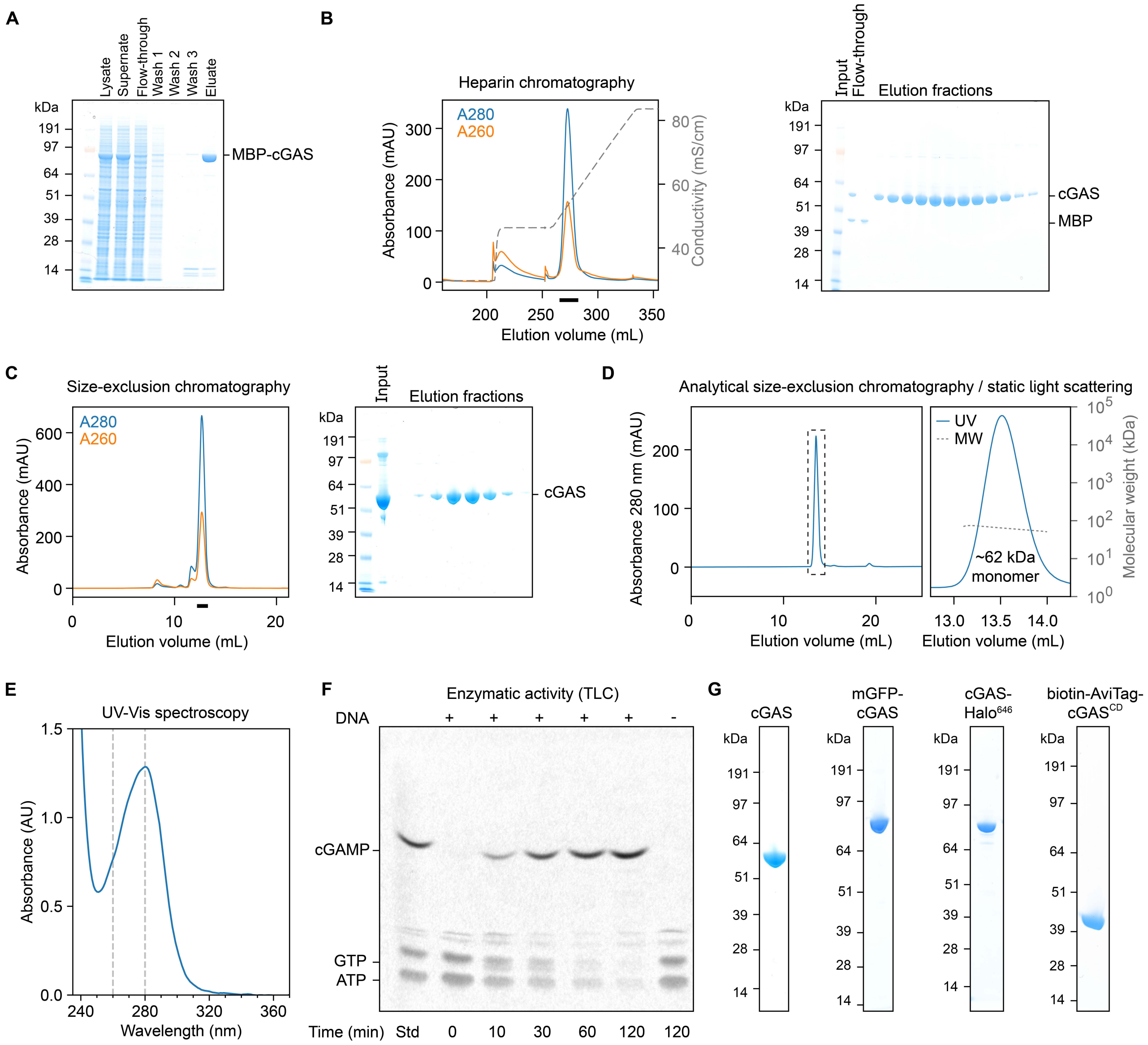

Fig. S1. Purification of full-length human cGAS from insect cells.

(**A**) Coomassie-stained SDS-PAGE gel of amylose affinity chromatography steps in MBP-cGAS purification. (**B**) Heparin affinity chromatography and Coomassie-stained SDS-PAGE gel of elution fractions. (**C**) Size-exclusion chromatography using a Superdex 200 Increase 10/300 column and Coomassie-stained SDS-PAGE gel of elution fractions. (**D**) Analytical size-exclusion chromatography using a Superdex 200 Increase 10/300 column, coupled to a static light scattering detector, confirmed that cGAS eluted as a single peak corresponding to a monomer. (**E**) Representative absorbance spectrum of recombinant cGAS. The A260/A280 absorbance ratio of 0.59 confirms protein purity. (**F**) Time course of cGAMP production by 1 µM cGAS upon the addition of 0.2 µM 100-bp DNA in reaction buffer containing 120 mM KCl, 20 mM HEPES, pH 7.5, 5 mM $MgCl_2$, 0.5 mM ATP, 0.5 mM GTP, 1 mM DTT, and 0.1 mg/mL BSA at 37 °C, analyzed by thin-layer chromatography. (**G**) Coomassie-stained SDS-PAGE gels of recombinant cGAS proteins used in this study.

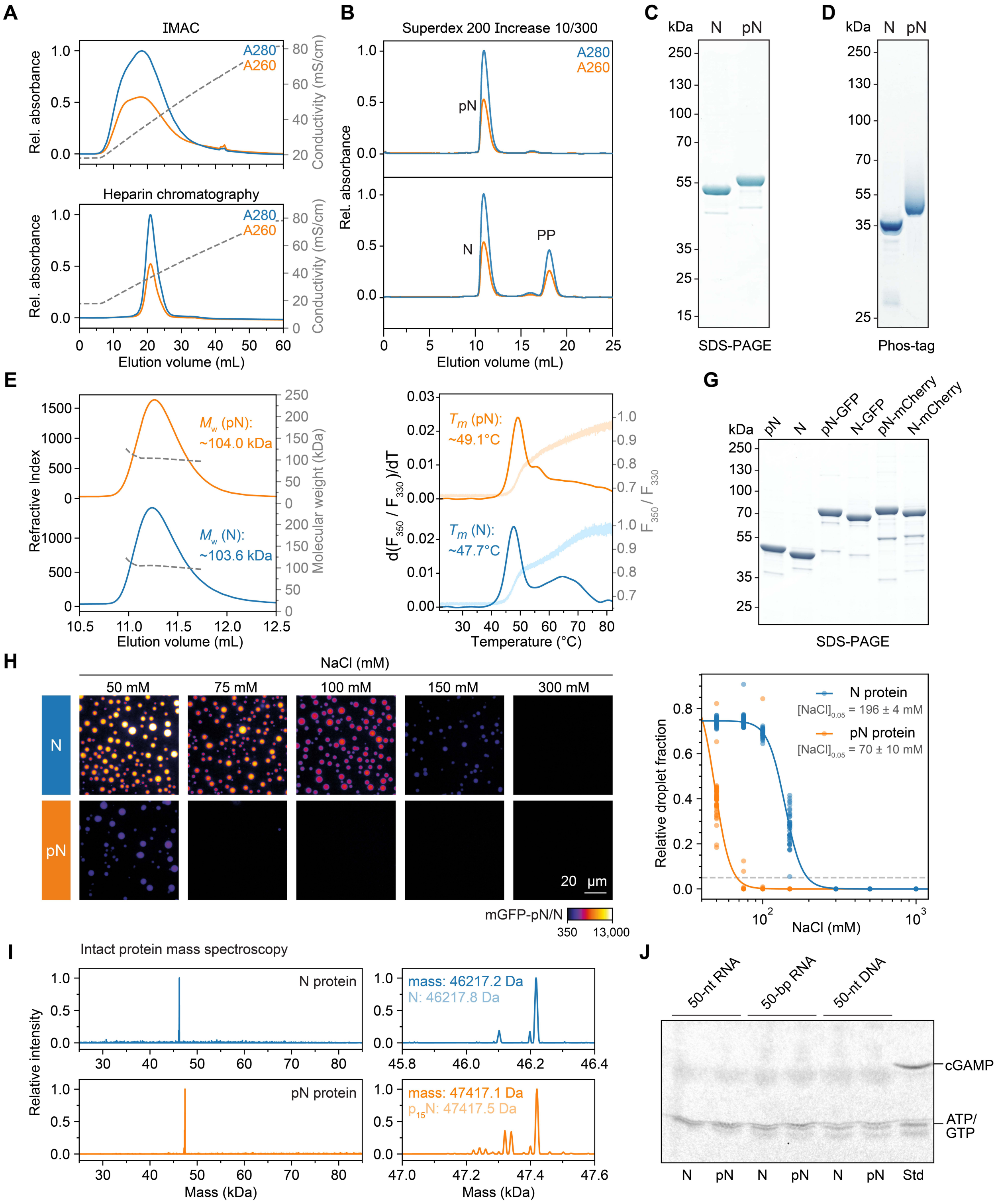

Fig. S2. Purification of full-length SARS-CoV-2 N and pN proteins.

(**A**) Immobilized metal affinity chromatography (IMAC, here nitrilotriacetic acid-immobilized Ni^{2+} ions) and heparin affinity chromatography traces. (**B**) Size-exclusion chromatography was

performed using a Superdex 200 Increase 10/300 column with hyperphosphorylated (pN) and dephosphorylated nucleocapsid protein (N) treated with λ-phosphatase (PP) prior to this chromatography step. (**C**) Coomassie-stained SDS-PAGE gel and (**D**) Phos-tag gel of purified N and pN proteins. (**E**) Analytical size-exclusion chromatography using a Superdex 200 Increase 10/300 column coupled to a static light scattering detector confirmed that pN and N proteins elute as a single peak corresponding to the molecular weight of a dimer. (**F**) Nano-differential scanning fluorimetry of pN and N proteins demonstrates the thermostability of both proteins. (**G**) Coomassie-stained SDS-PAGE gel of all nucleocapsid proteins used in this study. (**H**) Electrostatic contributions to homotypic nucleocapsid protein condensation. Condensate formation was monitored at a fixed concentration (10 μM) of N or pN protein while varying the ionic strength by NaCl titration in assay buffer (20 mM HEPES, pH 7.5, and 5 mM $MgCl_2$). The relative droplet fraction as a function of ionic strength is shown in the right panel. The dashed line denotes the arbitrarily defined condensation threshold. The corresponding critical sodium chloride concentrations ($[NaCl]_{0.05}$) are indicated. (**I**) Intact protein mass spectrometry of monomeric N (blue) and pN protein (orange) species, determined by HPLC-MS and SEC-MS, respectively. The right panels provide a detailed view of the major peaks. Experimentally obtained masses of the most abundant monomer species are indicated, with the theoretical masses shown below. Complete dephosphorylation of the N protein was confirmed, while the pN protein exhibits multiple proteoforms with up to 15 phosphorylation sites. Hyperphosphorylation of the nucleocapsid protein has been reported for SARS-CoV-2-infected mammalian cells (12-14) (**J**) Thin-layer chromatography analysis showing that cGAS is not activated by non-canonical ligands–Cy5-labeled 50-nt RNA, 50-bp RNA, or 50-nt DNA–in the presence of N or pN protein, unlike the canonical activator dsDNA. Conditions as in Fig. 3C.

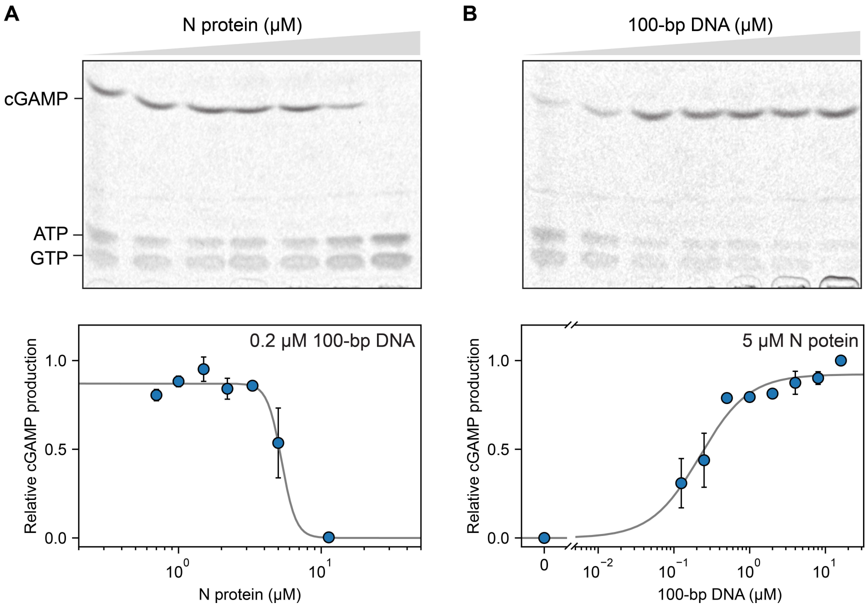

Fig. S3. Excess DNA reverses N protein inhibition of cGAS activity.
cGAMP synthesis by cGAS (1 µM) was assessed by thin-layer chromatography followed by densitometric quantification. Experiments were performed in a buffer containing 120 mM KCl, 20 mM HEPES, pH 7.5, 5 mM $MgCl_2$, 0.25 mM ATP, 0.25 mM GTP, and 1 mM DTT, upon the addition of N protein and DNA as specified below. (**A**) N protein titration in the presence of 0.2 µM 100-bp DNA. (**B**) 100-bp DNA titration in the presence of 5 µM N protein. The reactions were incubated for 4 h at 25 °C. Relative cGAMP production, normalized to the nucleocapsid-free, DNA-saturated control, is plotted. Data represent the mean ± standard deviation (n = 2 independent experiments).

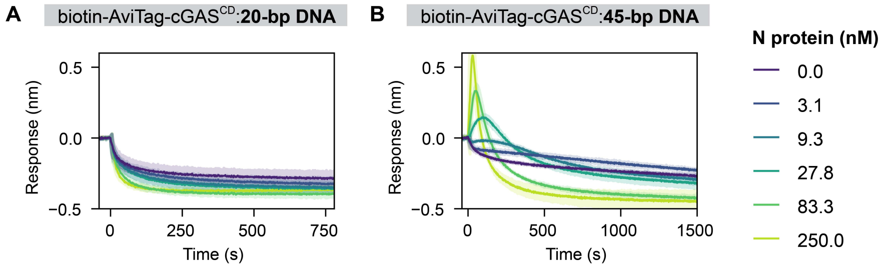

Fig. S4: The SARS-CoV-2 N protein accelerates DNA dissociation from cGAS.
Biotinylated AviTag-cGAS^{CD} was immobilized on a streptavidin sensor, and binding events were monitored via bio-layer interferometry (BLI). The sensor was subsequently exposed to a saturating concentration of (A) 20-bp DNA or (B) 45-bp DNA, followed by incubation with increasing concentrations of N protein. Experiments were conducted in a buffer containing 150 mM KCl, 20 mM HEPES, pH 7.5, and 0.05% (v/v) Tween-20. Data represent mean ± standard deviation (n = 2 independent experiments).

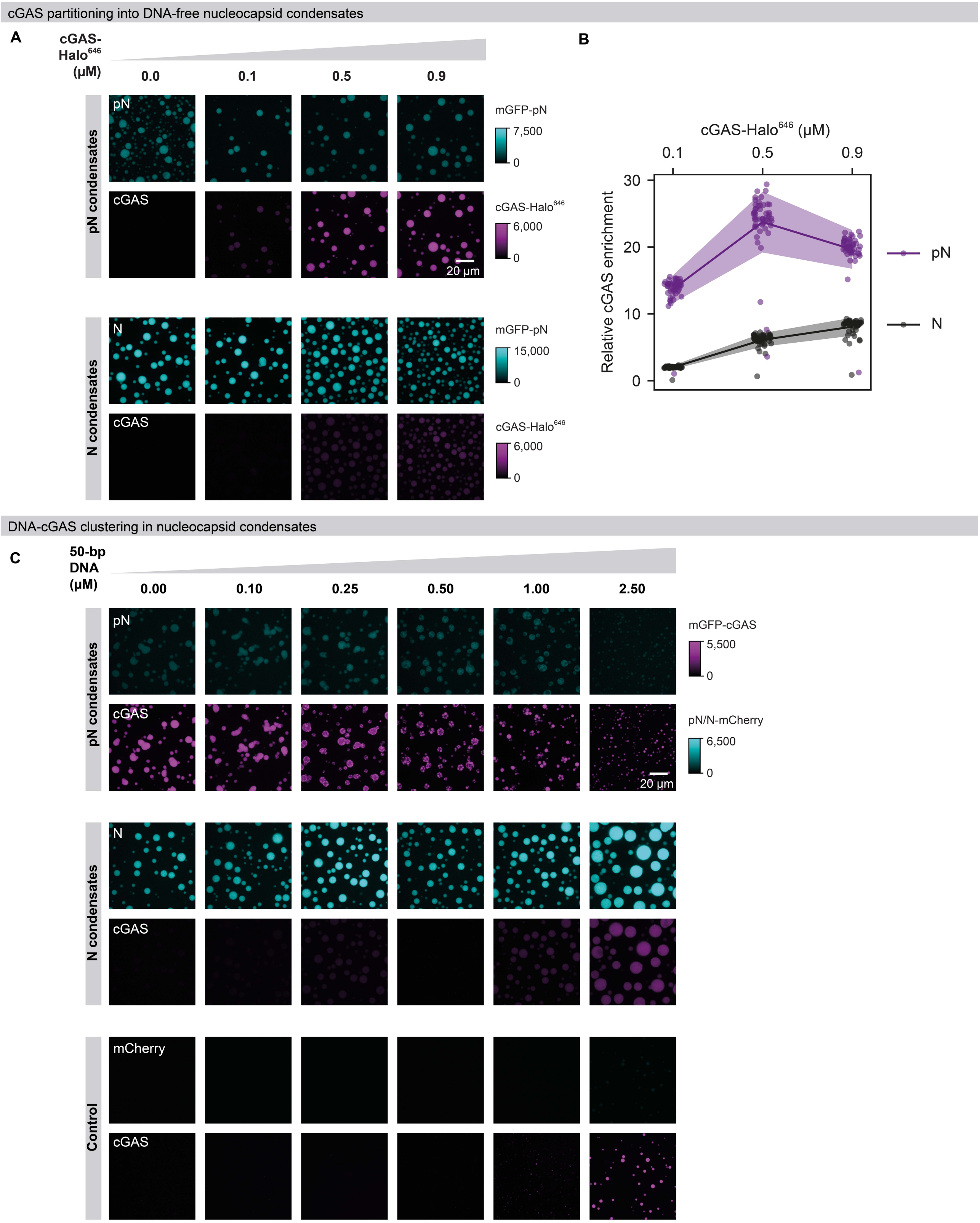

Fig. S5: cGAS partitioning in nucleocapsid protein condensates.

Condensates were assembled using SARS-CoV-2 nucleocapsid proteins (10 µM) in a buffer containing 50 mM NaCl, 20 mM HEPES, pH 7.5, 5 mM MgCl2, 5% glycerol, and 2 mM DTT, with cGAS and/or 50-bp DNA as indicated. (A) Titration of cGAS-Halo646 into DNA-free condensates formed with either pN (top) or N protein (bottom). (B) Quantification of cGAS enrichment in the dense phase demonstrates preferential partitioning into pN protein condensates in the absence of DNA. (C) Titration of 50-bp DNA to condensates in the presence of 1 µM cGAS-Halo646. cGAS partitions homogeneously into N protein condensates as DNA concentration increases. In pN condensates, cGAS demixes from the dense pN protein phase at DNA concentrations ≥ 250 nM. In the absence of nucleocapsid proteins, DNA–cGAS clusters become microscopically discernible at a DNA concentration of 1 µM. Notably, pN condensates facilitate the coarsening and clustering of nanoscopic DNA–cGAS assemblies, thereby promoting their coalescence and sedimentation. This results in increased detectability of DNA–cGAS clusters by light microscopy and an apparent overrepresentation of condensed cGAS–DNA relative to nucleocapsid protein-free samples.

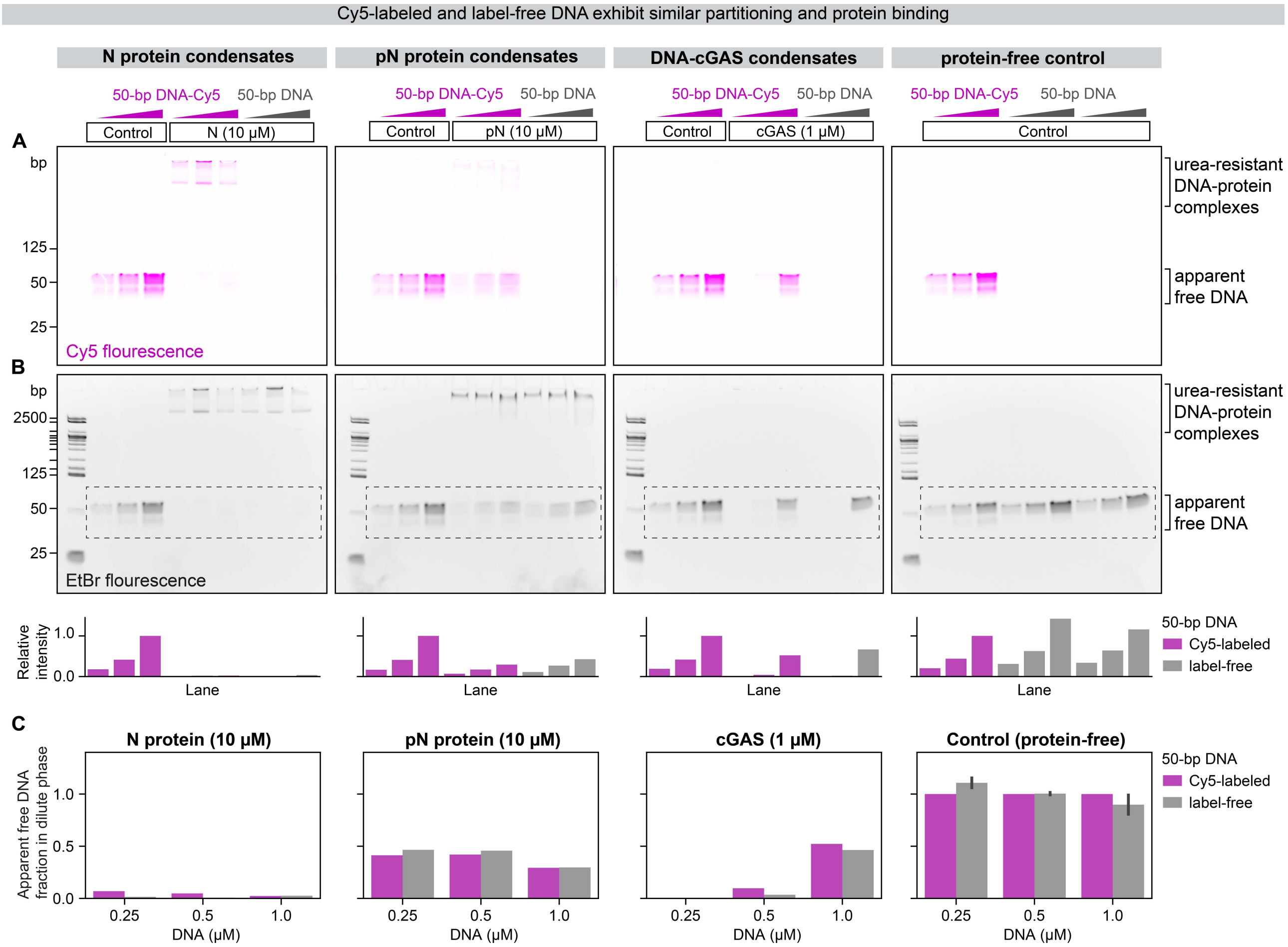

Fig. S6: Cy5 labeling does not affect DNA partitioning or urea sensitivity of DNA–protein complexes.

Condensates were formed using either SARS-CoV-2 N or pN protein (10 µM) or cGAS (1 µM) in the presence of Cy5-labeled or label-free 50-bp DNA at 0.25, 0.5, and 1.0 µM concentration in a buffer containing 20 mM HEPES, pH 7.5, 50 mM NaCl, 5 mM $MgCl_2$, 0.1 mM ATP, 0.1 mM GTP, 5% glycerol, and 2 mM DTT. Samples were incubated for 2 h before separating the dilute from the dense phase by centrifugation. Protein-free controls were processed analogously. (**A**, **B**) Denaturing Urea-PAGE of the dilute phase. DNA content of the dilute phase was analyzed by (**A**) Cy5-flourescence detection and (**B**) ethidium bromide staining. The 25-bp DNA ladder indicates bands corresponding to 2500, 2000, 1000, 700, 600, 500, 400, 300, 200, 150, 125, 50, and 25 bp (labels for 150-2000 bp are omitted for clarity). Urea-resistant DNA–protein complexes are indicated above. The apparent free DNA corresponds to the DNA remaining in the dilute phase after condensation. Dashed boxes highlight the apparent free DNA fraction used for quantification (below). (**C**) Quantification of the apparent free DNA fraction in the dilute phase relative to protein-free controls. The relative intensity of free Cy5-labeled (magenta) and label-free (gray) DNA was measured and normalized to DNA-only control samples.

Table S1. Recombinant proteins and plasmids used in this study.

Protein name	Plasmid ID	Plasmid name	Purified protein sequence (including linker, cloning, and cleavage site remnants)	Reference
cGAS	TH1823	pOCC102_pOEM1-His6-MBP-3C- cGAS	GPAAA-cGAS-GAP	This study
mGFP-cGAS	TH1824	pOCC131_pOEM1-His6-pfuMBP-3C-mGFP- cGAS -3C-His6	GP-mGFP-GSAGSAAGSGAAA-cGAS-GAP	This study
cGAS-Halo	TH2074	pOCC338_pOEM1-MBP-3C- cGAS-Halo	GPAAA-cGAS(1-522)-GAPGSAGSAAGSG-Halo	This study
AviTag-cGAS^{CD}	TH2111	pOCC517_pOEM1-MBP-3C-AviTag-cGAS(CD)	GP-AviTag-GSAGSAAGSGA-cGAS(158-522)-GAP	This study
N / pN protein	TH1750	pOCC102_pOEM1-His6-MBP-3C-NCAP[SARS2]	GPAAA-NCAP[SARS2]-GAP	(4)
mGFP-N / mGFP-pN protein	TH1749	pOCC175_pOEM1-His6-MBP-3C-mGFP-TEV-NCAP[SARS2]	GP-mGFP- GSSSGR-TEVsite-AAA-NCAP[SARS2]-GAP	(4)
N-mCherry / pN-mCherry protein	TH1883	pOCC216_pOEM1-MBP-3C-NCAP[SARS2]-Cherry-3C-His6	GPAAA-NCAP[SARS2]-GAPGSAGSAAGSG-Cherry-LEVLFQ	This study

Table S2. Oligonucleotides used in this study.

All oligos were purchased HPLC-purified. The 50-nt RNA and 50-bp DNA correspond to the 50-nt sequence from the CoV-2 lineage B genome 5' UTR (positions +45 to +94 in GenBank: MN908947.3), covering the 6-nt core transcriptional regulatory sequence (TRS, +70 to +75), which forms a stem loop and serves as a putative packaging sequence. 45-bp DNA and 100-bp DNA are derived from ISD (interferon stimulatory DNA), a 45-bp non-CpG oligomer from the *Listeria monocytogenes* genome (15).

Oligo name	Sequence (5'→3')	Supplier	Ref.
50-bp DNA-Cy5	forward:<br>Cy5-GATCTCTTGTAGATCTGTTCTCTAAACGAACTTTAAAATCTGTGTGGCTG<br>reverse:<br>CAGCCACACAGATTTTAAAGTTCGTTTAGAGAACAGATCTACAAGAGATC	IDT	(16)
50-bp DNA	forward:<br>GATCTCTTGTAGATCTGTTCTCTAAACGAACTTTAAAATCTGTGTGGCTG<br>reverse:<br>CAGCCACACAGATTTTAAAGTTCGTTTAGAGAACAGATCTACAAGAGATC	IDT	(16)
50-nt DNA-Cy5	Cy5-GATCTCTTGTAGATCTGTTCTCTAAACGAACTTTAAAATCTGTGTGGCTG	IDT	(16)
50-nt RNA-Cy5	Cy5-<br>rGrArUrCrUrCrUrUrGrUrArGrArUrCrUrGrUrUrCrUrCrUrArArArCrG<br>rArArCrUrUrUrArArArArUrCrUrGrUrGrUrGrGrCrUrG	IDT	(16)
50-bp RNA-Cy5	forward:<br>Cy5-<br>rGrArUrCrUrCrUrUrGrUrArGrArUrCrUrGrUrUrCrUrCrUrArArArCrG<br>rArArCrUrUrUrArArArArUrCrUrGrUrGrUrGrGrCrUrG<br>reverse:<br>rCrArGrCrCrArCrArCrArGrArUrUrUrUrArArArGrUrUrCrGrUrUrUrA<br>rGrArGrArArCrArGrArUrCrUrArCrArArGrArGrArUrC	IDT	(16)
45-bp DNA	forward:<br>TACAGATCTACTAGTGATCTATGACTGATCTGTACATGATCTACA<br>reverse:<br>TGTAGATCATGTACAGATCAGTCATAGATCACTAGTAGATCTGTA	Sigma-Aldrich	(15)
100-bp DNA	forward:<br>ACATCTAGTACATGTCTAGTCAGTATCTAGTGATTATCTAGACATACATCTAGTAC<br>ATGTCTAGTCAGTATCTAGTGATTATCTAGACATGGACTCATCC<br>reverse:<br>GGATGAGTCCATGTCTAGATAATCACTAGATACTGACTAGACATGTACTAGATGTA<br>TGTCTAGATAATCACTAGATACTGACTAGACATGTACTAGATGT	Sigma-Aldrich	(17)

SI References

1. R. P. Lemaitre, A. Bogdanova, B. Borgonovo, J. B. Woodruff, D. N. Drechsel, FlexiBAC: a versatile, open-source baculovirus vector system for protein expression, secretion, and proteolytic processing. *BMC Biotechnol* **19**, 20 (2019). http://doi.org/10.1186/s12896-019-0512-z.
2. E. Gasteiger *et al.*, "Protein Identification and Analysis Tools on the ExPASy Server" in The Proteomics Protocols Handbook, J. M. Walker, Ed. (Humana Press, Totowa, NJ, 2005), 10.1385/1-59259-890-0:571 chap. Chapter 52, pp. 571-607.
3. F. Wu *et al.*, A new coronavirus associated with human respiratory disease in China. *Nature* **579**, 265-269 (2020). http://doi.org/10.1038/s41586-020-2008-3.
4. W. E. Arter *et al.*, Biomolecular condensate phase diagrams with a combinatorial microdroplet platform. *Nat Commun* **13**, 7845 (2022). http://doi.org/10.1038/s41467-022-35265-7.
5. B. T. Kuhn *et al.*, "Biotinylation of membrane proteins for binder selections" in Expression, Purification, and Structural Biology of Membrane Proteins, C. Perez, T. Maier, Eds. (Springer US, New York, NY, 2020), 10.1007/978-1-0716-0373-4_11, pp. 151-165.
6. J. Schindelin *et al.*, Fiji: an open-source platform for biological-image analysis. *Nat Methods* **9**, 676-682 (2012). http://doi.org/10.1038/nmeth.2019.
7. T. A. Riley *et al.*, Synthesis of 2-(β -D-ribofuranosyl)pyrimidines, A new class of C-nucleosides. *Journal of Heterocyclic Chemistry* **24**, 955-964 (2009). http://doi.org/10.1002/jhet.5570240413.
8. J. Wang *et al.*, A Molecular Grammar Governing the Driving Forces for Phase Separation of Prion-like RNA Binding Proteins. *Cell* **174**, 688-699 e616 (2018). http://doi.org/10.1016/j.cell.2018.06.006.
9. J. Vanlier *et al.* (2024) lumicks/pylake: v1.5.3. http://doi.org/10.5281/zenodo.14008535.
10. E. C. Meng *et al.*, UCSF ChimeraX: Tools for structure building and analysis. *Protein Sci* **32**, e4792 (2023). http://doi.org/10.1002/pro.4792.
11. T. Gutmann, D. Kuster, A. A. Hyman, Source data for: SARS-CoV-2 nucleocapsid protein directly prevents cGAS-DNA recognition through competitive binding. Zenodo. https://doi.org/10.5281/zenodo.14475043. Deposited June 04 2025.
12. M. Bouhaddou *et al.*, The global phosphorylation landscape of SARS-CoV-2 infection. *Cell* **182**, 685-712 (2020). http://doi.org/10.1016/j.cell.2020.06.034.
13. K. Klann *et al.*, Growth Factor Receptor Signaling Inhibition Prevents SARS-CoV-2 Replication. *Mol Cell* **80**, 164-174 e164 (2020). http://doi.org/10.1016/j.molcel.2020.08.006.
14. A. D. Davidson *et al.*, Characterisation of the transcriptome and proteome of SARS-CoV-2 reveals a cell passage induced in-frame deletion of the furin-like cleavage site from the spike glycoprotein. *Genome Med* **12**, 68 (2020). http://doi.org/10.1186/s13073-020-00763-0.
15. D. B. Stetson, R. Medzhitov, Recognition of cytosolic DNA activates an IRF3-dependent innate immune response. *Immunity* **24**, 93-103 (2006). http://doi.org/10.1016/j.immuni.2005.12.003.
16. D. Yang, J. L. Leibowitz, The structure and functions of coronavirus genomic 3' and 5' ends. *Virus Res* **206**, 120-133 (2015). http://doi.org/10.1016/j.virusres.2015.02.025.
17. L. Andreeva *et al.*, cGAS senses long and HMGB/TFAM-bound U-turn DNA by forming protein-DNA ladders. *Nature* **549**, 394-398 (2017). http://doi.org/10.1038/nature23890.
